# Genome-wide A-to-I RNA editing in fungi independent of ADAR enzymes

**DOI:** 10.1101/gr.199877.115

**Published:** 2016-04

**Authors:** Huiquan Liu, Qinhu Wang, Yi He, Lingfeng Chen, Chaofeng Hao, Cong Jiang, Yang Li, Yafeng Dai, Zhensheng Kang, Jin-Rong Xu

**Affiliations:** 1 State Key Laboratory of Crop Stress Biology for Arid Areas, Purdue-NWAFU Joint Research Center, College of Plant Protection, Northwest A&F University, Yangling, Shaanxi 712100, China;; 2 College of Life Sciences, Northwest A&F University, Yangling, Shaanxi 712100, China;; 3 Department of Botany and Plant Pathology, Purdue University, West Lafayette, Indiana 47907, USA

## Abstract

Yeasts and filamentous fungi do not have adenosine deaminase acting on RNA (ADAR) orthologs and are believed to lack A-to-I RNA editing, which is the most prevalent editing of mRNA in animals. However, during this study with the *PUK1* (FGRRES_01058) pseudokinase gene important for sexual reproduction in *Fusarium graminearum*, we found that two tandem stop codons, UA^1831^GUA^1834^G, in its kinase domain were changed to UG^1831^GUG^1834^G by RNA editing in perithecia. To confirm A-to-I editing of *PUK1* transcripts, strand-specific RNA-seq data were generated with RNA isolated from conidia, hyphae, and perithecia. *PUK1* was almost specifically expressed in perithecia, and 90% of transcripts were edited to UG^1831^GUG^1834^G. Genome-wide analysis identified 26,056 perithecium-specific A-to-I editing sites. Unlike those in animals, 70.5% of A-to-I editing sites in *F. graminearum* occur in coding regions, and more than two-thirds of them result in amino acid changes, including editing of 69 *PUK1*-like pseudogenes with stop codons in ORFs. *PUK1* orthologs and other pseudogenes also displayed stage-specific expression and editing in *Neurospora crassa* and *F. verticillioides*. Furthermore, *F. graminearum* differs from animals in the sequence preference and structure selectivity of A-to-I editing sites. Whereas A's embedded in RNA stems are targeted by ADARs, RNA editing in *F. graminearum* preferentially targets A's in hairpin loops, which is similar to the anticodon loop of tRNA targeted by adenosine deaminases acting on tRNA (ADATs). Overall, our results showed that A-to-I RNA editing occurs specifically during sexual reproduction and mainly in the coding regions in filamentous ascomycetes, involving adenosine deamination mechanisms distinct from metazoan ADARs.

RNA editing is a post-transcriptional event that recodes hereditary information by changing the nucleotide sequence of RNA molecules (Farajollahi and Maas 2010; Rosenthal 2015). Although various RNA editing systems that target major types of cellular RNA have been identified in eukaryotes (Farajollahi and Maas 2010; Knoop 2011; Gray 2012), only two types of RNA editing are known to cause changes in nuclear-encoded messenger RNA (mRNA). One is the cytidine-to-uridine (C-to-U) RNA editing that is rare in mammals, such as the well-characterized target of *APOB* mRNA in human intestine (Powell et al. 1987). The other is the adenosine-to-inosine (A-to-I) editing, the most prevalent type of RNA editing known in the animal kingdom (Bass 2002).

A-to-I RNA editing occurs when A residues are converted to I residues via an enzymatic deamination reaction. Whereas editing of mRNA is mediated by the adenosine deaminase acting on RNA (ADAR) family of enzymes that convert A to I in double-stranded RNA (dsRNA) substrates (Bass 2002; Savva et al. 2012), the adenosine deaminase acting on tRNA (ADAT) enzymes act on transfer RNAs (tRNAs) (Keller et al. 1999; Torres et al. 2014). Because I is subsequently recognized as guanosine (G) by the translation machinery, A-to-I substitution in coding regions (CDSs) of mRNA may lead to codon changes and alter functional properties of proteins (Maas 2010; Nishikura 2010). However, editing events resulting in protein sequence recoding are not common in animals. To date, the majority of studies on the functional consequences of recoding editing are related to genes important for animal nervous systems (Hood and Emeson 2012; Rosenthal 2015). A-to-I editing of ligand- and voltage-gated ion channels and neurotransmitter receptors in invertebrates (Patton et al. 1997; Palladino et al. 2000) and vertebrates (Sommer et al. 1991; Seeburg 1996) are among the best-studied ADAR-mediated recoding events. More recently, A-to-I editing of K^+^ channels responding to temperature adaptation also has been reported in octopuses (Garrett and Rosenthal 2012). Despite these findings, the biological functions of mRNA editing in animals remains poorly understood because the majority of A-to-I editing events occur in the noncoding regions.

By RNA-seq analyses, a large number of A-to-I RNA editing events have been identified in the transcriptomes of humans (Li et al. 2009; Bahn et al. 2012; Peng et al. 2012; Ramaswami et al. 2013; Sakurai et al. 2014) and other animals (Danecek et al. 2012; St Laurent et al. 2013; Chen et al. 2014; Alon et al. 2015). In general, RNA editing occurs more frequently in noncoding than in coding regions. In humans, more than 1.4 million A-to-I editing sites have been identified (Pinto et al. 2014). The vast majority (∼97%) of them target repetitive sequences located within introns and 5′- or 3′-untranslated regions (UTRs) (Ramaswami and Li 2014). Only a small fraction of these editing sites (approximately 100) occur in the coding regions and result in amino acid changes. Similar observations have been reported in other animals. Although most editing sites do not affect the primary sequences of coding proteins, only approximately 50 recoding events in mice (Danecek et al. 2012), 645 in *Drosophila* (St Laurent et al. 2013), and eight in *Caenorhabditis elegans* (Zhao et al. 2015) have been identified. One remarkable exception among animals that have been studied for A-to-I editing is squid, in which a total of 57,108 recoding RNA editing sites were identified in its nervous system (Alon et al. 2015), raising the question about the prevalence of recoding RNA editing events in other animal lineages (Rosenthal 2015).

ADATs and A-to-I editing on tRNA are found in all domains of life (Torres et al. 2014). To date, however, ADARs and A-to-I editing of mRNA are only found in metazoans and is thought to be a metazoan innovation (Jin et al. 2009; Grice and Degnan 2015). Yeasts and filamentous fungi do not have ADAR orthologs in their genomes and are believed to lack A-to-I mRNA editing. However, during this study with a protein kinase named Puk1 (Perithecium unique kinase 1) (gene ID: FGRRES_01058, FG01058.1, or FGSG_01058), we found that the two tandem stop codons, UAG UAG, in its ORF were changed to UGG UGG by RNA editing in *Fusarium graminearum*, a causal agent of Fusarium head blight (FHB) of wheat and barley (Bai and Shaner 2004; Goswami and Kistler 2004). *F. graminearum* is a haploid homothallic ascomycete that overwinters in infected plant debris as saprophytic hyphae and produces perithecia and ascospores in the spring. Ascospores are forcibly discharged and dispersed by wind as the primary inoculum for FHB (Schmale et al. 2005; Trail 2009). We then conducted RNA-seq analysis to confirm the editing events of *PUK1* transcripts during sexual reproduction and identify genome-wide A-to-I editing events in *F. graminearum*. In addition, to analyze characteristics of mRNA editing sites in *F. graminearum*, we also identified A-to-I RNA editing events in *Neurospora crassa* and *F. verticillioides*. Furthermore, we attempted to experimentally characterize biological functions of A-to-I RNA editing in late stages of sexual reproduction and identify responsible deaminase genes in *F. graminearum*.

## Results

### The Puk1 kinase plays a stage-specific role during sexual reproduction

**Figure 1.**
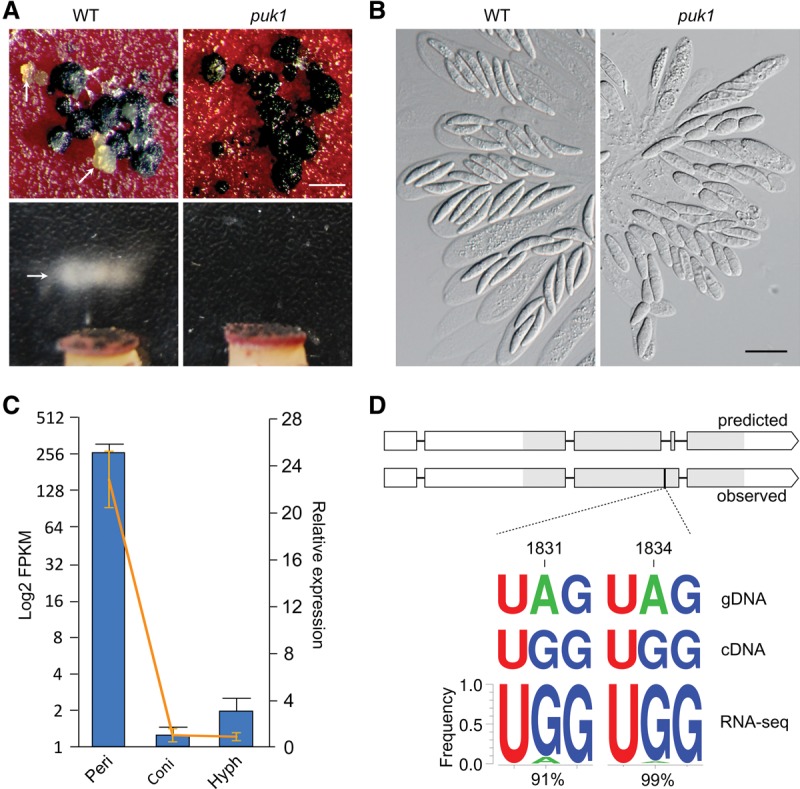
Function, expression, and RNA editing of *PUK1*. (*A*) Mating cultures of the wild-type PH-1 (WT) and *puk1* mutant were examined for cirrhus production (*upper*) and ascospore release (*lower*). Arrows point to cirrhi (ascospores oozing) and ascospore masses ejected from perithecia. Bar, 1 mm. (*B*) Asci and ascospores formed by PH-1 and the *puk1* mutant. Deletion of *PUK1* affected ascospore morphology. Bar, 20 μm. (*C*) The expression level of *PUK1* in conidia (Coni), 24-h hyphae (Hyph), and perithecia collected at 8 dpf (Peri). The bar chart represents the absolute expression level (log_2_ FPKM) in RNA-seq data, and the line is the relative expression level (2^−ΔΔCt^) assayed by qRT-PCR (the expression level of *PUK1* in conidia arbitrarily set to one). Error bars indicate standard deviation calculated from two biological replicates of RNA-seq data or three biological replicates for qRT-PCR. (*D*) The gene structure and editing sites of *PUK1.* The gene model and coding region of *PUK1* is different between automated annotation (predicted) and actual cDNA sequence (observed). Rectangle boxes are coding regions, and the protein kinase domain region is in gray. The corrected gene model contains two tandem stop codons, UA^1831^G UA^1834^G (1830–1835, marked with a black vertical line) in its coding region that is part of an intron introduced erroneously by automated annotation. A^1831^and A^1834^ in the genomic DNA (gDNA) were changed to G's in cDNA sequences by RNA editing. WebLogo shows the frequency of A-to-G variants at each site in RNA-seq reads.

The Puk1 protein kinase is conserved in filamentous fungi but lacks a distinct ortholog in the budding and fission yeasts. Two *puk1* deletion mutants generated in our previous study of the *F. graminearum* kinome (Wang et al. 2011) were confirmed by Southern blot analysis in this study. All the *puk1* mutants were normal in growth, conidiation, and plant infection but were defective in late stages of sexual reproduction. Close examinations showed that perithecia formed by the mutant were normal in size and morphology but defective in ascospore release and cirrhus formation (Fig. 1A). Whereas normal ascospores were four-celled, *puk1* ascospores were often single- or two-celled and spherical or fragmented (Fig. 1B). When assayed by qRT-PCR, *PUK1* transcription was barely detectable in conidia and vegetative hyphae but significantly up-regulated in perithecia collected at 8 d post-fertilization (dpf) (Fig. 1C). These results suggest that *PUK1* has stage-specific expression and functions during sexual reproduction.

### A-to-G variants in *PUK1* cDNA

To identify Puk1-interacting proteins in *F. graminearum* by yeast two-hybrid assays, we generated the bait construct of *PUK1* with its ORF amplified from the first-strand cDNA synthesized with RNA isolated from mating cultures of the wild-type strain PH-1 harvested at 8 dpf. To our surprise, sequencing analysis showed that all the Puk1 bait constructs contained the third predicted intron, which is in the ninth subdomain of the kinase domain (Fig. 1D). Interestingly, the sequence of this putative intron in the Puk1 bait construct differed from its genomic DNA at nucleotides 1831 and 1834 (Fig. 1D). At both positions, A is in the genome sequence but G is in the yeast two-hybrid bait construct.

To verify this observation, we sequenced the *PUK1* fragments amplified from genomic DNA and cDNA. Whereas A^1831^ and A^1834^ were in the PCR products amplified from genomic DNA, G^1831^ and G^1834^ were in cDNA fragments, indicating that the third intron of *PUK1* is incorrectly predicted and these two nucleotides may be subjected to A-to-I editing in *F. graminearum*. Although it has not been reported in fungi, A-to-I RNA editing is a well-known phenomenon in animals.

### More than 90% of *PUK1* transcripts have the A-to-I editing events at A^1831^ and A^1834^

We then conducted RNA-seq analysis with RNA isolated from conidia, 24-h hyphae, and 8-dpf perithecia of PH-1. For each fungal tissue, strand-specific RNA-seq data were generated by Illumina sequencing with 150-bp paired-end reads for two independent biological replicates. For each tissue, approximately 70 million high quality reads were obtained (Supplemental Table 1).

Only rare *PUK1* transcripts were present in RNA-seq data of conidia and hyphae (fragments per kilobases of exons per million mapped reads [FPKM] <2) and none of them had G^1831^ or G^1834^ (Supplemental Fig. 1). For the *PUK1* reads present in RNA-seq data of perithecia, >99% of them had either G^1831^ or G^1834^. Of these, 90% had both G^1831^ and G^1834^ (Fig. 1D). These results indicate that A-to-I RNA editing of *PUK1* transcripts occurred at a high frequency in perithecia.

To determine the effects of A-to-I editing on *PUK1* expression and function, we generated the *PUK1*^TGGTGG^ allele with the TA^1831^G TA^1834^G sequence changed to TG^1831^G TG^1834^G and the *PUK1*^TAATAA^ allele with two tandem stop codons TAA TAA added behind the TA^1831^G TA^1834^G sequence. Both mutant alleles were transformed into the *puk1* deletion mutant. In the resulting transformants, both *PUK1*^TGGTGG^ and *PUK1*^TAATAA^, similar to the wild-type *PUK1* allele, had relatively low expression levels in vegetative hyphae (Supplemental Fig. 2A). In comparison with *PUK1*^WT^, the expression level of *PUK1*^TGGTGG^ was increased ∼1.7-fold, whereas *PUK1*^TAATAA^ expression was slightly reduced (Supplemental Fig. 2A). These results indicate that the promoter of *PUK1* but not the editing events plays a major role in its up-regulated expression during sexual reproduction. Because the editing sites TG^1831^G TG^1834^G are in the conserved protein kinase domains, it is not surprising that expression of *PUK1*^TAATAA^ failed to complement the defects of the *puk1* mutant in ascospore morphology and release (Supplemental Fig. 2B,C). Therefore, A-to-I editing is essential for *PUK1* to be functional during sexual reproduction.

### A-to-I RNA editing occurs specifically during sexual reproduction

**Figure 2.**
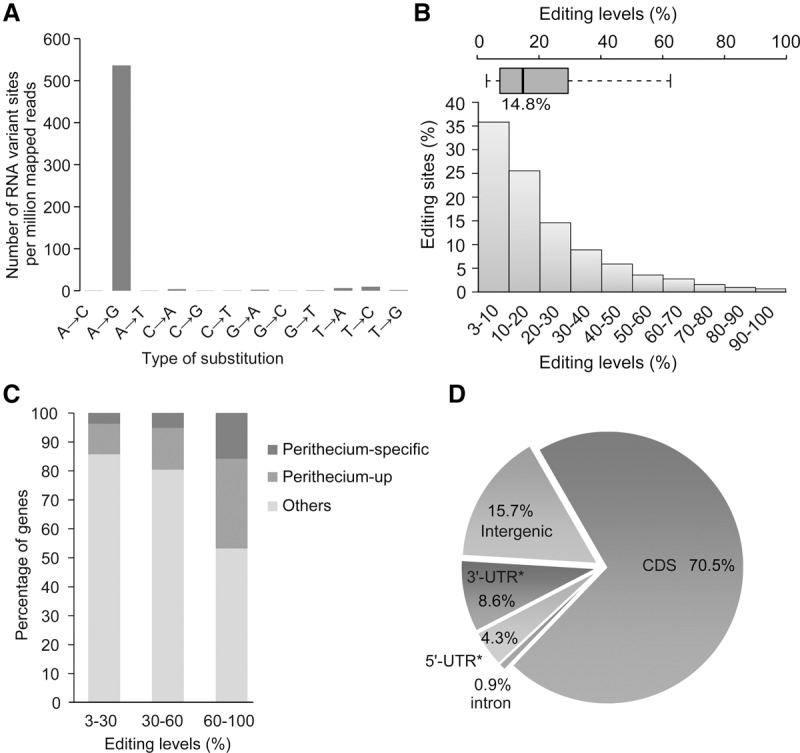
Properties of the A-to-I editing sites in *F. graminearum*. (*A*) The number of each type of RNA variant (gDNA → cDNA) sites per million mapped unduplicated reads identified in the two RNA-seq data of 8-dpf perithecia. (*B*) Histogram and box plot showing the frequency of RNA editing levels. The majority of editing sites have editing levels <30%. (*C*) The percentage of marked categories of genes that have editing sites of different editing levels. Genes that were specifically expressed (Perithecium-specific) or up-regulated (Perithecium-up) in perithecia were identified by comparative analysis of RNA-seq data of conidia, hyphae, and 8-dpf perithecia (see Supplemental Methods). (*D*) The distribution of 26,056 A-to-I editing sites. Because only a few genes have known UTRs in *F. graminearum*, we used the 500-bp region upstream of the start codon and the 500-bp region downstream from the stop codon to represent the 5′- and 3′-UTRs, respectively.

We then performed a genome-wide identification of A-to-I RNA editing events in *F. graminearum*. In comparison with genomic sequences, 27,301 single-nucleotide variant (SNV) sites were identified in the combined RNA-seq data of two independent biological replicates of 8-dpf perithecia (Fig. 2A; Supplemental Table 2). Strikingly, 26,056 of these SNVs (>95%) correspond to the A-to-G transition, which is consistent with A-to-I editing. Assuming that non-A-to-G variants (1245) are false positives and the error rate for all replacement types is equal, the estimated false-discovery rate in our A-to-G identification is only 0.43%, which is 10-fold lower than the error rate of A-to-I editing analysis in humans and animals (Bahn et al. 2012; Peng et al. 2012; Ramaswami et al. 2013; Sakurai et al. 2014; Alon et al. 2015). These results showed that genome-wide A-to-I editing (A-to-G SNV) occurs in *F. graminearum*.

We also conducted a similar analysis with RNA-seq data of conidia and hyphae. In total, only 68 and 112 A-to-G SNV sites were identified in conidia and hyphae, respectively (Supplemental Fig. 3; Supplemental Table 2). In comparison with 335 and 452 non-A-to-G SNV sites, no enrichment for A-to-G SNV sites was observed in RNA-seq data of conidia and hyphae, indicating that A-to-I editing specifically occurs during sexual reproduction.

### Genes with sites of higher editing levels tend to be up-regulated or specifically expressed in perithecia

We then examined the RNA editing level for each A-to-I editing site as the percentage of reads with the A-to-G variant among all the reads covering that site. Similar to what have been reported in humans, rhesus macaque, and flies (Li et al. 2009; Bahn et al. 2012; Ramaswami et al. 2013; Chen et al. 2014), the A-to-I editing level varied from 3% to 100% among different editing sites, with the median level of 14.8% in *F. graminearum* (Fig. 2B). Among all 26,056 A-to-G sites in perithecia, 75.9% had editing levels <30%, whereas only 5.8% had editing levels >60%. However, we noticed that genes with editing sites of higher editing levels were enriched for genes likely important for sexual reproduction (Fig. 2C). Among the 10,652 genes expressed in perithecia, 971 (9.1%) were specifically expressed, and 1256 (11.8%) were up-regulated at least twofold during sexual reproduction in comparison with conidia and hyphae. Approximately 47% of the genes with editing sites of >60% A-to-I editing levels were either specifically expressed or up-regulated at least twofold in perithecia compared to conidia and hyphae. In contrast, only 14% of the genes with editing sites of editing levels <30% had increased expression levels during sexual reproduction (Fig. 2C). These results suggest that A-to-I editing occurs more efficiently in genes that display stage-specific or unregulated expression in perithecia in *F. graminearum.* Nevertheless, the median FPKM values for genes with editing sites of <30%, 30%–60%, and >60% editing levels are 31.5, 25.5, and 27.5, respectively. Therefore, there is no general correlation between expression levels and editing levels in *F. graminearum*.

### The majority of A-to-I editing events occur in the CDSs in *F. graminearum*

In humans and *Drosophila*, A-to-I editing primarily occurs in introns and UTRs (Bahn et al. 2012; Park et al. 2012; St Laurent et al. 2013; Sakurai et al. 2014). When the distribution of 27,301 A-to-I editing sites was analyzed, to our surprise, 21,095 of them (70.5%) are in the CDSs in *F. graminearum* (Fig. 2D). Only 0.9%, 12.9%, and 15.7% of A-to-I editing sites were in intronic, UTR, and intergenic regions, respectively. Therefore, unlike in metazoans, A-to-I editing may preferentially occur in the CDSs in fungi. Nevertheless, A-to-I editing sites appear to occur more frequently in 3′-UTRs (8.6%) than in 5′-UTRs (4.3%) in *F. graminearum*, which is similar to observations in humans and *Drosophila* (Bahn et al. 2012; Peng et al. 2012; St Laurent et al. 2013; Zhang and Xiao 2015).

### More than two-thirds of the editing events in CDSs result in amino acid changes

**Figure 3.**
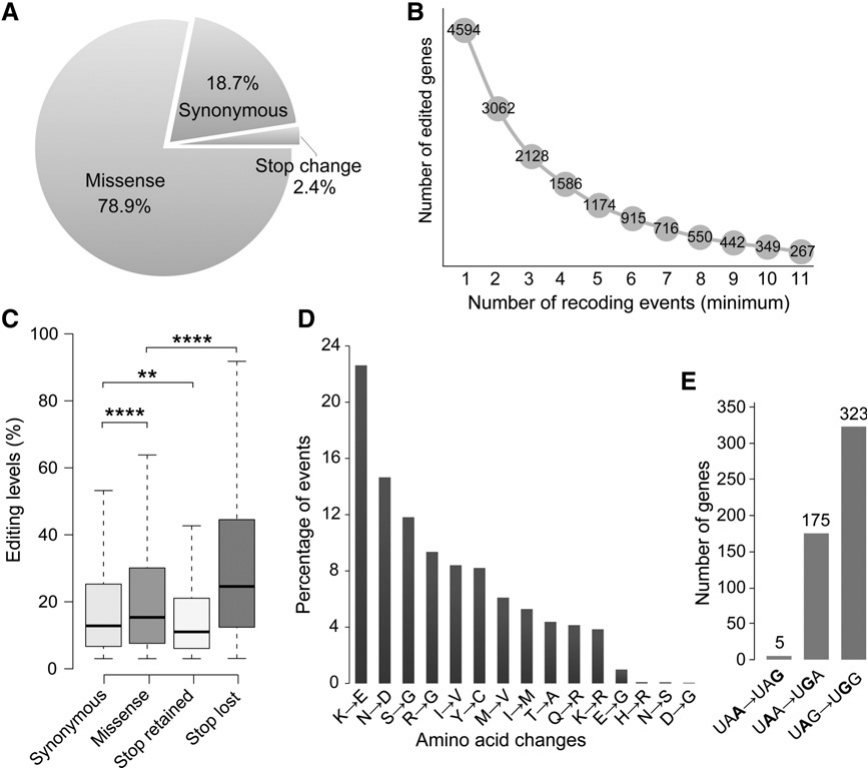
Functional consequences of the A-to-I editing sites in *F. graminearum.* (*A*) The percentage of editing events resulting in different types of changes in protein sequences or coding regions. The stop change category includes stop-loss and stop-retained editing events. (*B*) The number of genes with different numbers of recoding A-to-I editing events. (*C*) Box plots showing the editing levels of RNA editing sites with different types of functional consequences. The statistical significance (*t*-test) for each comparison is indicated: (****) *P* < 0.0001; (**) *P* < 0.01. (*D*) Percentage of missense A-to-I editing events resulting in different types of amino acid changes. (*E*) Numbers of genes with marked stop-loss or stop-retained RNA editing events. The nucleotides subjected to RNA editing are in bold.

Among the 21,095 A-to-I editing sites in CDSs, 78.9% (16,649) of them are missense editing events that result in amino acid substitutions (Fig. 3A). In contrast, only 18.7% are synonymous editing. In total, 4594 of 10,652 (43%) protein coding genes expressed in perithecium samples (more than 10 counts per million) have at least one missense editing event (Fig. 3B). Almost half of them (2128) harbor three or more recoding sites. Transcripts of 349 genes (8%) contain at least 10 recoding sites. Two genes with the most abundant recoding events are FGRRES_06089 and FGRRES_08133 that have 38 and 37 editing sites, respectively. FGRRES_08133 is an essential gene in *F. graminearum* (Wang et al. 2011). It encodes a 2395-aa protein kinase orthologous to the Tor1 and Tor2 of the budding yeast that are involved in regulating various biochemical and cellular processes, including transcription, translation, autophagy, and meiosis (Martin and Hall 2005). FGRRES_06089 encodes a 3880-aa protein that is orthologous to Tra1, a subunit of the yeast SAGA and NuA4 histone acetyltransferase complexes that are important for gene regulation and DNA repair (Doyon et al. 2004; Rodríguez-Navarro 2009). Interestingly, Tra1, Tor1, and Tor2 all are members of the PIKK (phosphoinositide three-kinase-related kinase) family.

Furthermore, the editing level of missense editing sites (median, 15.4%) is statistically significantly higher than that of synonymous editing sites (median, 12.8%) (Fig. 3C). Notably, 76.2% (12,694) of the missense editing sites resulted in changes to amino acid residues of different physicochemical properties, including 22% lysine (K) (basic) to glutamate (E) (acidic), 14% asparagine (N) (nonacidic) to aspartate (D) (acidic), and 11% serine (S) (polar) to G (nonpolar) changes (Fig. 3D). These observations suggest that A-to-I editing may be important for adaptation and diversification protein functions during sexual reproduction in *F. graminearum*.

### Editing events in transcripts of 503 genes occur at the stop codon

Interestingly, none of the A-to-I editing events resulted in changes from amino acid codons to stop codons (nonsense change), suggesting that the editing mechanism avoids generation of truncated proteins in *F. graminearum*. However, the stop codons of 503 genes were edited (Fig. 3E). Among these, 323 had the stop-loss events, in which UAG was changed to the UGG tryptophan codon, resulting in the addition of an extra stretch of amino acid residues to the C-terminal end of predicted protein sequences. Although the biological significance of stop-loss editing is not clear, transcripts of 11 transcriptional factor and five protein kinase genes known to be important or essential for sexual reproduction in *F. graminearum* (Son et al. 2011; Wang et al. 2011; Li et al. 2015; Liu et al. 2015) are among the genes with stop-loss A-to-I editing (Supplemental Table 3). Moreover, the editing level of stop-loss events is significantly higher than that of other editing events (Fig. 3C), suggesting selection toward read-through of these proteins.

For the other 180 editing events that occurred at stop codons, 175 had the UAA to UGA change and five had the UAA to UAG change. In general, UAA is the most efficient stop codon, whereas UGA is a relative leaky stop codon that sometimes causes read-through in bacteria, fungi, and mammals (Jin et al. 2002; Stiebler et al. 2014). Therefore, even for these edited transcripts with stop codons retained, A-to-I editing likely increases the read-through frequency.

### An additional 69 sexual reproduction-related genes have similar editing events with *PUK1*

To search for genes with similar editing events to *PUK1*, we manually examined editing sites that resided in the predicted introns. Sixty-nine of them had the UAG to UGG change in exons that were erroneously annotated as intronic sequences by automated annotation based on our RNA-seq data (Supplemental Table 4). Therefore, *PUK1* is not the only gene in *F. graminearum* with stop codons in the coding regions that are subjected to stage-specific RNA editing but avoided by automated annotation with incorrectly predicted introns.

Among these 69 genes with *PUK1*-like editing events, 54 of them (78%) were specifically expressed or up-regulated in perithecia, including the *rid* (RIP defective) ortholog (FGRRES_08648) and genes encoding putative Dcp1-like mRNA-decapping enzyme (FGRRES_02091), Rho GTPase (FGRRES_01649), and Rho-GAP protein (FGRRES_08999). However, a majority of them (Supplemental Table 4) have no known functional protein domains, and their functions during sexual reproduction remain to be identified. Nevertheless, for two of them, FGRRES_10728 and FGRRES_01563, their orthologs in the budding yeast are important for meiosis. Although FGRRES_10728 is orthologous to yeast *AMA1* that encodes an activator of meiotic anaphase promoting complex (Cooper et al. 2000), FGRRES_01563 is an ortholog of yeast *SPO22* that encodes a meiosis-specific protein essential for chromosome synapsis (Primig et al. 2000). It is likely that A-to-I RNA editing of these two genes during sexual reproduction is important for meiosis in *F. graminearum*.

We then selected five of them, FGRRES_01649, FGRRES_ 10094, FGRRES_08389, FGRRES_12623_M, and FGRRES_14031_ M (Supplemental Table 4), that encode hypothetical proteins in *F. graminearum* for functional characterization and identified at least three knockout mutants for each gene. None of the deletion mutants of FGRRES_08389, FGRRES_12623_M, and FGRRES_ 14031_M had obvious defects in growth and sexual reproduction (Supplemental Fig. 4). Deletion of FGRRES_10094 had no effects on hyphal growth, perithecium development, and ascospore formation. However, the FGRRES_10094 deletion mutant was defective in ascospore release, and the ascus wall became dissolved in 12-dpf perithecia. When 12-dpf perithecia were cracked open, only aggregates of ascospores were observed in the mutant, but fascicles of asci were observed in the wild type (Supplemental Fig. 5). Yellowish ascospore cirrhi were rarely observed, and massive discharging of ascospores from perithecia was not observed in the FGRRES_10094 deletion mutant (Supplemental Fig. 5). Deletion of FGRRES_01649 that encodes a putative Rho GTPase resulted in defects in ascospore formation and release. Perithecia formed by the FGRRES_01649 mutant were normal in morphology but produced and released fewer mature ascospores (Supplemental Fig. 5). These results showed that both FGRRES_10094 and FGRRES_01649 play roles in the late stages of sexual development.

### A-to-I editing has strong sequence preference in *F. graminearum*

**Figure 4.**
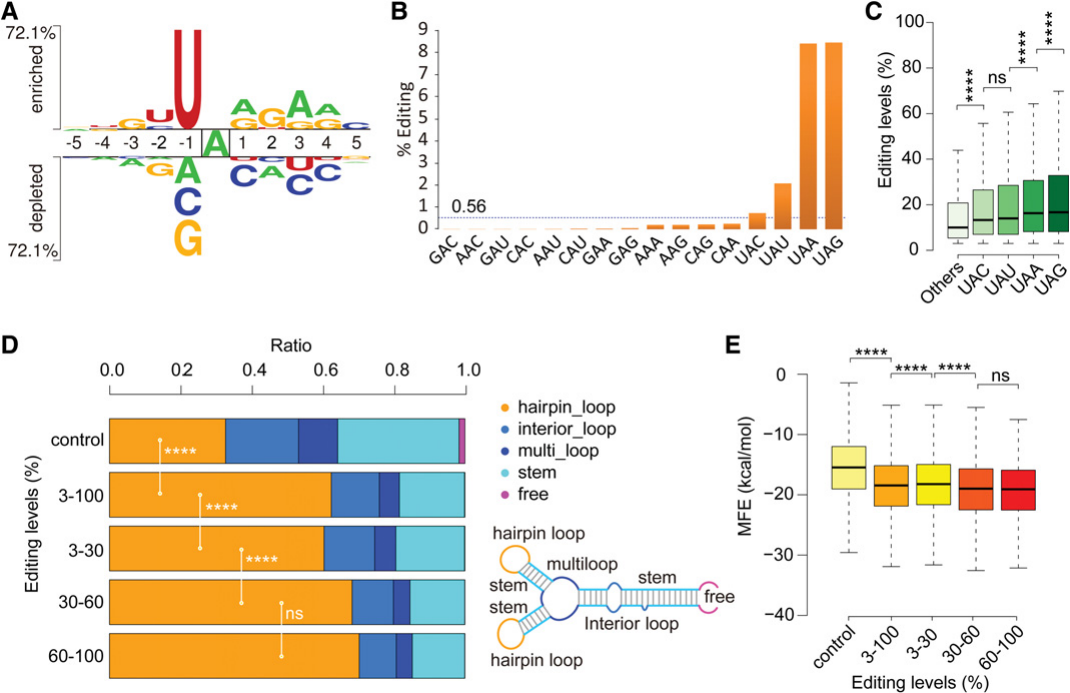
Sequence and structure preferences of the A-to-I editing sites in *F. graminearum*. (*A*) Two Sample Logo showing the enriched (*above* the *top* line) and depleted (*below* the *bottom* line) nucleotides nearby the A's targeted for RNA editing (*P* < 0.01, *t*-test), with the level of preference or depletion proportional to the scale. A total of 30,000 adenosine sites randomly chosen from predicted cDNA sequences were used as the negative control. (*B*) The percentage of marked triplet sequences with A-to-I editing events. For each of the 16 possible triplets centered on the edited adenosine (NAN), the number of observed editing events was divided by its total occurrence in cDNA sequences. The horizontal dotted line marks the average percentage (0.56%) of editing events observed in these 16 NAN triplets. (*C*) Box plot comparing the editing levels of editing sites in different triplets: (****) *P* < 0.0001, *t*-test; (ns) not significant. (*D*) Stacked column showing the ratio of RNA editing events of marked editing levels in the five types of RNA secondary structure elements diagrammed on the *right*. The predicted RNA secondary structure is based on 30-nt upstream and 30-nt downstream sequences surrounding the edited A's. The statistical significance for hairpin loop ratio comparison is indicated: (****) *P* < 0.0001, χ^2^-test; (ns) not significant. (*E*) Box plot showing the minimum free energy (MFE) of predicted hairpin loops with A-to-I editing events of different editing levels. The statistical significance for each comparison is indicated: (****) *P* < 0.0001, *t*-test; (ns) not significant.

When the flanking sequences of all the A-to-I editing sites identified in this study were analyzed, the 5′ − 1 site preference is U (85.74%) > A (8.03%) > G (4.98%) > C (1.25%). Although the overall trend is similar to that of human ADAR (also known as ADAR1) and ADARB1 (also known as ADAR2) (U > A > C > G) (Eggington et al. 2011), the significant enrichment of U at the −1 position is unique to *F. graminearum* (Fig. 4A). The 3′ +1 site preference is A (39.16%) ≈ G (36.92%) > U (17.00%) > C (6.93%), which is different from that of human ADAR (G > C ≈ A > U) or ADARB1 (G > C > U ≈ A) (Eggington et al. 2011). In *F. graminearum*, A and G are enriched at the +1 position of edited A sites (Fig. 4A).

When the percentage of edited triplets was estimated for all 16 possible triplets centered on edited A's (NAN), we found that almost 9% of UAG or UAA have the A-to-I editing events (Fig. 4B), making these two preferred triplet sequences for RNA editing in *F. graminearum*. In contrast, triplets such as GAC, AAC, GAU, and CAC are rarely edited (Fig. 4B). Although the editing preference of UAG triplets has been reported in studies with metazoan ADARs (Li et al. 2009; Eggington et al. 2011; Kuttan and Bass 2012; St Laurent et al. 2013; Sakurai et al. 2014), the preference for UAA triplets appears to be unique to *F. graminearum*. Furthermore, we noticed that the preferred triplets tend to have higher editing levels (Fig. 4C). Indeed, the median editing level for the non-UAN triplets is only ∼10% but is >16% for the UAG and UAA triplets (Fig. 4C).

In addition, we observed a weak base preference at the positions beyond the nearest neighboring nucleotides (Fig. 4A). Whereas the −2 and −3 positions are slightly enriched for U and G, respectively, the +2 position is enriched for G. An overrepresentation of A and G at both the +3 and +4 positions also observed (Fig. 4A). These findings suggest that A-to-I editing is influenced by more than the −1 and +1 nucleotides of the edited A's, particularly by the downstream nucleotides.

### A-to-I editing is highly structure selective in *F. graminearum*

Because RNA structure is known to affect editing specificity (Barraud and Allain 2012), we predicted the secondary structures of RNA sequences containing the A-to-I editing sites identified in this study. Interestingly, 62.4% of the editing sites were in hairpin loops (Fig. 4D). Furthermore, the percentage of editing sites in hairpin loops appears to increase for those with higher editing levels. For edited A sites with 60% or higher editing levels, >70% of them are in hairpin loops (Fig. 4D). These results suggest that A-to-I editing preferentially occurs to A's in hairpin loops in *F. graminearum*, which is different from preferred editing sites in RNA stems in animals (Barraud and Allain 2012).

In addition, we found that the minimum free energy (MFE) of predicted hairpin loops with A-to-I editing sites is significantly lower than that of random controls (Fig. 4E). Furthermore, sites with 60% or higher editing levels tend to be in hairpin loops with lower MFEs than those with <30% editing levels (Fig. 4E). These results indicate that RNA editing selectively targets structurally stable hairpin loops, and the stability of hairpin loops affects editing efficiency in *F. graminearum*.

### None of the ADAT genes in *F. graminearum* is specifically expressed during sexual reproduction

The *F. graminearum* genome contains no predicted genes that encode proteins with both adenine deaminase domain and dsRNA binding domain, the hallmark of ADARs (Nishikura 2010; Savva et al. 2012). In fact, none of the fungal species that have been sequenced have putative ADAR genes. Therefore, like yeasts and other filamentous fungi, *F. graminearum* lacks distinct ADAR orthologs and may involve other proteins for A-to-I editing.

**Figure 5.**
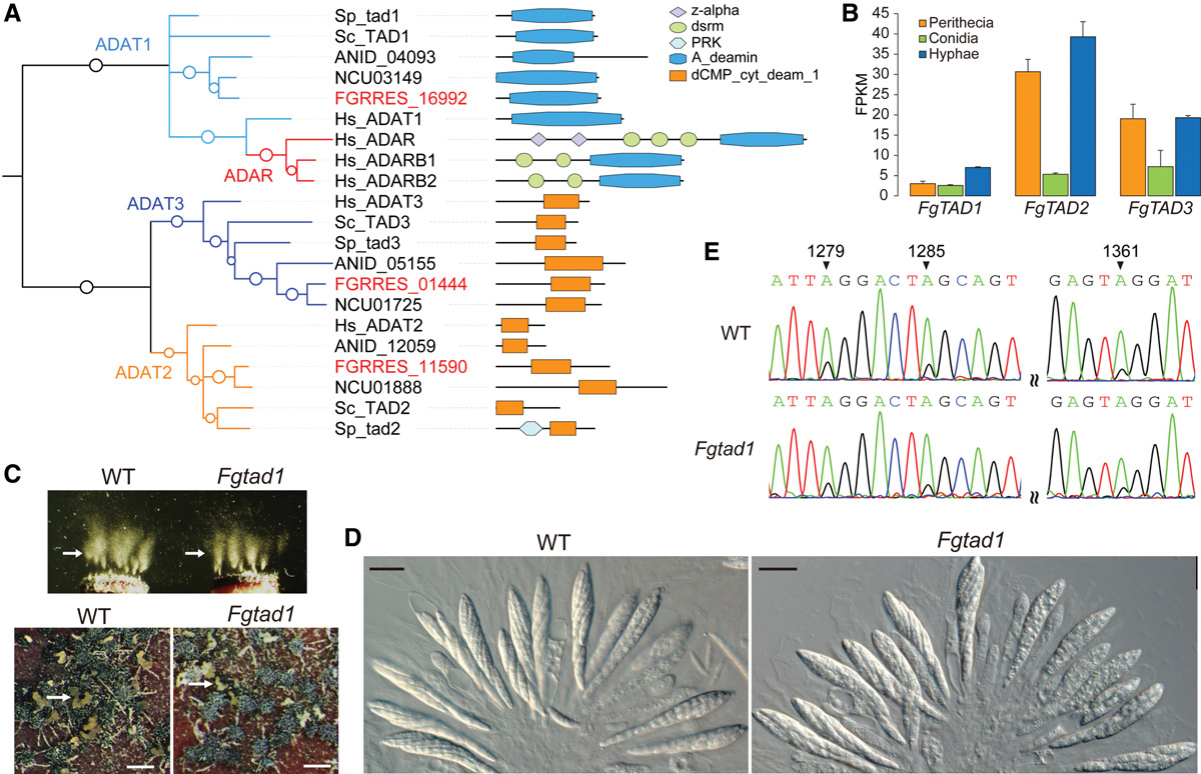
Evolution, expression, and function of ADATs in *F. graminearum*. (*A*) Phylogenetic tree of deaminase domain of fungal ADATs and ADARs constructed using PhyML3.1 (Guindon et al. 2010). The SH-like support of approximate likelihood ratios (aLRT-SH) is plotted as circles on the branches (only SH-like support >0.6 are shown). The prefixes for gene names or IDs are as follows: (ANID) *Aspergillus nidulans*; (FGRRES) *Fusarium graminearum*; (Hs) *Homo sapiens*; (NCU) *Neurospora crassa*; (Sc) *Saccharomyces cerevisiae*; (Sp) *Schizosaccharomyces pombe*. The domain structures are as follows: (A_deamin) adenosine-deaminase (PF02137); (dCMP_cyt_deam_1) cytidine and deoxycytidylate deaminase zinc-binding region (PF00383); (dsrm) double-stranded RNA binding motif (PF00035); (PRK) phosphoribulokinase (PF00485); (z-alpha) adenosine deaminase z-alpha domain (PF02295). (*B*) The expression level (fragments per kilobases of exons for per million mapped reads [FPKM]) of three ADAT genes of *F. graminearum* estimated with RNA-seq data of conidia, 24-h hyphae, and perithecia collected at 8 dpf. Error bars indicate standard deviations calculated from two biological replicates of RNA-seq data. (*C*) Mating cultures of the wild-type PH-1 (WT) and *Fgtad1* deletion mutant were examined for ascospore release and cirrhus production. Arrows point to ascospore masses and cirrhi (ascospores oozing) ejected from perithecia. Bar, 1 mm. (*D*) Asci and ascospores formed by PH-1 and the *Fgtad1* mutants were examined with 12-dpf perithecia. Bar, 20 μm. (*E*) Sequencing traces for the edited region of *FgSSN3* (FGRRES_04484) amplified from RNA isolated from perithecia of PH-1 and *Fgtad1* mutant. Black arrows mark the edited A's that have a mixed peak of A and G in sequencing traces.

A-to-I editing in *F. graminearum* preferentially targets A's in hairpin loops, which is similar to the anticodon loop of tRNA targeted by ADATs, implying a potential evolutionary link between mRNA editing and ADATs in fungi. The *F. graminearum* genome has three predicted ADAT genes, FGRRES_16992, FGRRES_ 11590, and FGRRES_01444, that are orthologous to yeast *TAD1*, *TAD2*, and *TAD3*, respectively (Fig. 5A). Although FgTad1 contains only an adenosine-deaminase domain, both FgTad2 and FgTad3 have a cytidine/deoxycytidylate deaminase domain (Fig. 5A). Based on our RNA-seq data, none of these three ADAT genes were specifically expressed during sexual reproduction. The expression level of *FgTAD1* was relatively low under all the conditions assayed, but *FgTAD2* and *FgTAD2* had higher expression levels in hyphae and perithecia than in conidia (Fig. 5B), suggesting that *FgTAD2* and/or *FgTAD3* may play a more important role in A-to-I editing in perithecia in *F. graminearum*.

To determine their functions in mRNA editing, we attempted to generate ADAT knockout mutants by targeted gene replacement (Catlett et al. 2003). Five *Fgtad1* deletion mutants were identified in 10 hygromycin-resistant transformants that were screened, which is consistent with earlier publications of high gene replacement efficiency in *F. graminearum* (Son et al. 2011; Wang et al. 2011). Nevertheless, we failed to identify putative *Fgtad2* or *Fgtad3* mutants after screening hundreds of transformants generated in at least three different transformations, suggesting that deletion of *FgTAD2* or *FgTAD2* may be lethal. For the five *Fgtad1* mutants, none had any obvious defects in perithecium formation and ascosporogenesis or ascospore release (Fig. 5C,D). When assayed by RT-PCR and sequencing analysis of transcripts of the *FgSSN3* gene (FGRRES_04484), A-to-I editing events still occurred in perithecia of the *Fgtad1* mutants (Fig. 5E). These results suggest that *FgTAD1* is dispensable for A-to-I editing during sexual reproduction.

### RNA editing also occurs to *PUK1* orthologs in *Neurospora crassa* and *F. verticillioides*

**Figure 6.**
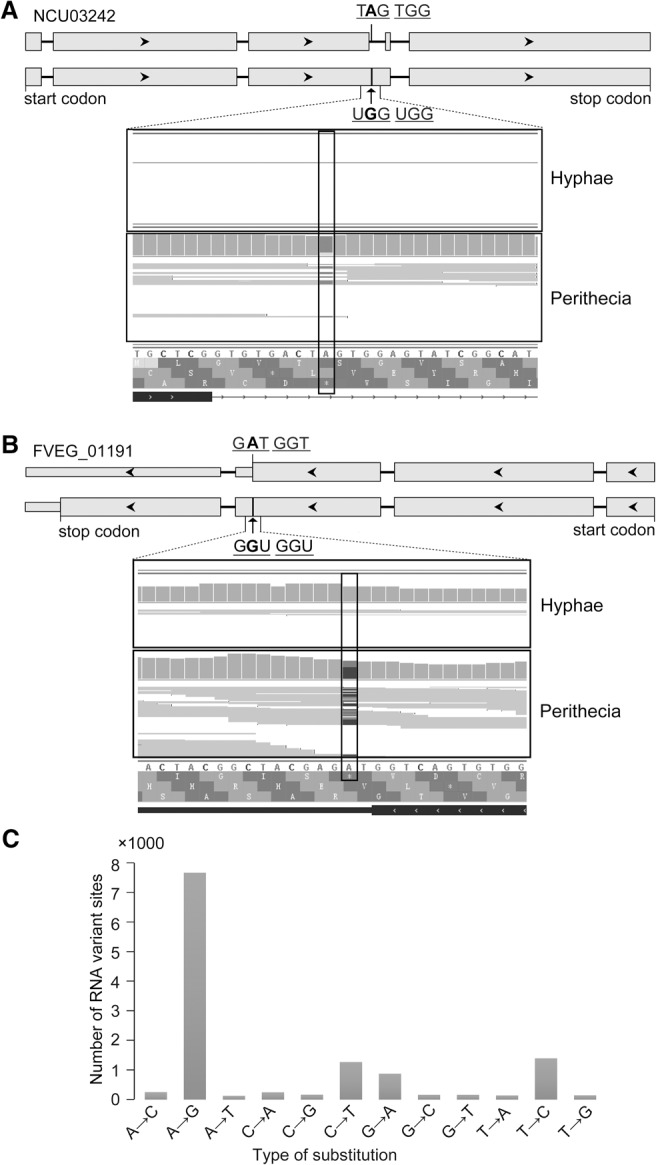
A-to-I RNA editing in *Neurospora crassa* and *Fusarium verticillioides*. RNA editing in transcripts of the *PUK1* orthologs in *N. crassa* (*A*) and *F. verticillioides* (*B*). The gene structure based on automated annotation (*upper*) differs from the actual cDNA sequence (*lower*) identified by RNA-seq analysis. Rectangle boxes are coding regions, and black arrowheads indicate the direction. In *N. crassa*, NCU03242 is specifically expressed in perithecia, and its UA^1628^G stop codon (marked with a black vertical line) corresponding to UA^1831^G of *PUK1* was edited to UGG. In *F. verticillioides*, RNA editing of UA^1836^G in FVEG_01191 (black vertical line) corresponding to UA^1834^G of *PUK1* was also only observed in perithecia. (*C*) Numbers of labeled RNA variant sites identified in RNA-seq data of perithecia in *F. verticillioides*.

*PUK1* is a protein kinase gene conserved in *N. crassa* and other Sordariomycetes. The predicted gene NCU03242 of *N. crassa* has a UAG stop codon in the kinase domain that is equivalent to the first of two tandem stop codons in the *PUK1* ORF (Fig. 6A). Comparative analysis of published RNA-seq data (Wang et al. 2014) revealed that the A-to-I editing event at this site (UAGUGG to UGGUGG) also occurs in the transcripts of NCU03242 in perithecia (Fig. 6A). Similar to *PUK1*, the transcription level of NCU03242 was relatively low, and A-to-I editing events were not observed in vegetative hyphae and other developmental stages in *N. crassa* (Wang et al. 2014).

Interestingly, the *PUK1* ortholog in *F. verticillioides* (FVEG_ 01191) has a stop codon that is equivalent to the second of two tandem stop codons in its ORF. Based on published RNA-seq data (Sikhakolli et al. 2012), A-to-I editing also occurs at this site and results in the UGGUAG to UGGUGG change in FVEG_ 01191 transcripts in perithecia but not in hyphae (Fig. 6B). These results indicate that stage-specific A-to-I editing also occurs in *N. crassa* and *F. verticillioides* during sexual reproduction.

### Genome-wide A-to-I RNA editing also occurs in *F. verticillioides*

We then analyzed genome-wide A-to-I editing sites in *F. verticillioides* (Ma et al. 2010) with published RNA-seq data (Supplemental Table 1; Sikhakolli et al. 2012). In *F. verticillioides*, a total of 7659 A-to-I editing sites were identified in RNA-seq data of perithecia with an estimated false discovery rate of 5.7% (Fig. 6C). Among them, 1685 (22%) are conserved in *F. graminearum.* No obvious enrichment for A-to-G variants was observed in the RNA-seq data of hyphae (Supplemental Fig. 6). Therefore, stage-specific A-to-I RNA editing occurs during sexual reproduction in *F. verticillioides* and possibly many other Sordariomycetes. In comparison with that of *F. graminearum*, the number of A-to-I editing sites identified in *F. verticillioides* is relatively small, which is due to the limitation of the RNA-seq data that are currently available and more stringent filters used during our analysis for the latter (see Supplemental Methods).

Furthermore, for the other 69 *F. graminearum* genes with *PUK1*-like editing events, 47 have orthologs in *F. verticillioides* (Supplemental Table 4). Among them, 34 (72%) also had the same editing events that resulted in stop codon to amino acid codon changes in perithecia in *F. verticillioides* (Supplemental Table 4). Because *F. graminearum* and *F. verticillioides* are closely related to each other, it is likely that these orthologous genes with the same stage-specific editing events have similar functions during sexual development.

## Discussion

According to cDNA clone sequencing and RNA-seq data, the gene model of *PUK1* based on automated annotation is incorrect with the third intron. However, the corrected coding region of *PUK1* contains two tandem stop codons UA^1831^G UA^1834^G in the kinase domain, which codes for a truncated, nonfunctional protein kinase. It appears that automated annotation introduces an intron to avoid these two stop codons that are changed to UG^1831^G UG^1834^G after RNA editing. Besides *PUK1*, we identified at least 69 other genes that have introns erroneously introduced by automated annotation to avoid in-frame stop codons that are subjected to A-to-I editing for the UAG to UGG change during sexual reproduction. Without RNA editing in hyphae or conidia, these genes are pseudogene-like because of the stop codons in their ORFs. Although the truncated proteins encoded by these pseudogene-like genes are likely nonfunctional in other stages, our findings suggest that they may have an important biological function during sexual reproduction. To our knowledge, this kind of A-to-I RNA editing events have not been functionally characterized or reported in humans, *Drosophila*, and other animals.

In this study, a total of 26,056 A-to-I editing sites were identified in *F. graminearum*, which is the first report of genome-wide A-to-I editing of mRNAs outside the animal kingdom. Interestingly, >70% of the editing events occur in coding regions and more than two-thirds of them are missense editing events, resulting in amino acid changes in 4594 *F. graminearum* genes. Although a similar finding has been reported recently in squid (Alon et al. 2015), A-to-I editing mainly occurs in noncoding regions, including introns and UTRs, in other animals (Bahn et al. 2012; Park et al. 2012; St Laurent et al. 2013; Sakurai et al. 2014). Furthermore, >76% of the missense editing sites in *F. graminearum* resulted in changing to amino acids of different physicochemical properties, which is different from squid (Alon et al. 2015). Therefore, unlike in metazoans, A-to-I editing tends to diversify protein functions in *F. graminearum*. If RNA editing also occurs in other fungi, predicted proteins based on automated annotation for genes subjected to A-to-I editing may display stage-specific differences.

Another unique feature of RNA editing in *F. graminearum* is that both the frequency and editing levels of nonsynonymous A-to-I editing sites are significantly higher than those of synonymous editing sites. Therefore, although RNA editing events are generally not advantageous in humans (Xu and Zhang 2014), A-to-I editing of mRNA may be subjected to selection for new traits or functions in fungi. We also noticed that *F. graminearum* has transcripts of more than 300 genes with the stop-loss editing events, including a number of transcription factor and kinase genes known to be important or essential for sexual reproduction (Son et al. 2011; Wang et al. 2011; Li et al. 2015; Liu et al. 2015). The large number of stop-loss editing events is somewhat unique to *F. graminearum*. Thus, it will be important to determine the biological functions of editing events resulting in the read-through of UAG stop codons.

Last, but not the least striking feature of A-to-I editing in *F. graminearum* is its stage-specific occurrence during sexual reproduction. Unlike in perithecium samples, no real RNA editing events were identified in RNA-seq data of conidia and hyphae. In animals, A-to-I RNA editing has been identified in virtually all the tissues examined. Nevertheless, A-to-I editing is known to have some tissue or developmental stage preference and is more frequent in brains than in other tissues (Paul and Bass 1998; Valente and Nishikura 2005; Li and Church 2013). Because ascospores (sexual spores) are forcibly discharged and dispersed by wind as the primary inoculum for this important pathogen (Schmale et al. 2005; Trail 2009), RNA editing during sexual reproduction may provide additional flexibility or variations in protein coding genes in this important pathogen. Approximately 47% of the genes with >60% editing levels are specifically expressed or up-regulated in perithecia, confirming the specific role of RNA editing during sexual reproduction in *F. graminearum.* At least three of these genes with stage-specific editing events, *PUK1*, FGRRES_ 10094, and FGRRES_01649, were functionally characterized to be important for ascospore formation and release, which is a critical step in the wheat head blight disease cycle. In *N. crassa*, deletion of *STK-21* (NCU03242), the ortholog of *PUK1*, did not result in obvious defects in sexual reproduction (Park et al. 2011). Whereas *F. graminearum* is homothallic, *N. crassa* is a heterothallic fungus. Park and colleagues only crossed the *stk-21* mutant with a wild-type strain (*stk-21* × *STK-21*^WT^) (Park et al. 2011), and it remains possible that the *stk-21* × *stk-21* mutant crosses may be defective in sexual reproduction.

For *PUK1*, its orthologs in *N. crassa* and *F. verticillioides* also are subjected to A-to-I editing at the same sites during sexual reproduction. Interestingly, the two A's in the UAGUAG sequence have been changed to G's in the genomic sequence of *PUK1* orthologs in some Sordariomycetes such as *Magnaporthe oryzae* and *Claviceps paspali*. In metazoans, it has been reported that the genomic G-to-A mutation may be corrected by A-to-I RNA editing, whereas the edited I (G) may be fixed into the genome sequences during evolution (Tian et al. 2008). For the 47 genes orthologous to *F. graminearum* genes with *PUK1*-like editing events, 34 of them have similar editing sites in *F. verticillioides*. The other 13 genes have these editing sites that may function to correct the G-to-A DNA mutations that occurred specifically in *F. graminearum* or be fixed in the *F. verticillioides* genome during evolution. Furthermore, genome-wide A-to-I RNA editing events also were identified in RNA-seq data of perithecia in *F. verticillioides*. Therefore, it is likely that stage-specific A-to-I editing is a common phenomenon in some filamentous ascomycetes during sexual reproduction. RNA editing may play an important role in regulating the functions of genes important for ascosporogenesis or forcible ascospore release.

A-to-I RNA editing in animals is mediated by members of the ADAR family that contain a conserved C-terminal catalytic deaminase domain and a variable number of N-terminal dsRNA binding domains (dsRBDs) (Nishikura 2010; Savva et al. 2012). In humans, the deaminase activities of ADAR and ADARB1 but not ADARB2 (also known as ADAR3) have been established by in vitro assays. Unlike the other two that are expressed in most human tissues, ADARB2 is specifically expressed in the central nervous system and it lacks a dsRBD (Chen et al. 2000). To date, ADAR genes have been identified in nearly all metazoans from sponges to humans, but not in plants, yeasts, or filamentous fungi (Grice and Degnan 2015). Indeed, we failed to identify ADAR genes in *F. graminearum*, *F. verticillioides*, and *N. crassa*. The lack of distinct ADAR orthologs in filamentous fungi suggests that the enzymes and related molecular mechanisms responsible for the A-to-I editing are different between fungi and animals.

However, *F. graminearum* has three putative ADAT genes. In eukaryotic organisms, ADAT enzymes specifically catalyze the deamination of adenosines to inosines at or adjacent to the tRNA anticodon (Gerber et al. 1998; Gerber and Keller 1999; Bass 2002; Keegan et al. 2004). They have a single adenosine deaminase domain that is closely related to that of ADARs but lack the dsRBD. Nevertheless, it appears that the dsRBD of ADARs is not essential for A-to-I editing although important for the deamination rate (Eggington et al. 2011). When overexpressed in *S. cerevisiae*, the deaminase domain of human ADARB1 is capable of binding to dsRNA and displays A-to-I editing activities independent of its dsRBD (Eifler et al. 2013). Given the sequence similarities and phyletic distributions, it has been proposed that the ADAR enzymes are a metazoan innovation that may have evolved from an ADAT ancestor via the addition of the dsRBD (Bass 2002; Grice and Degnan 2015). Some of the ADAT enzymes may have the stage-specific activity to edit mRNAs in *F. graminearum* and other filamentous ascomycetes, although in vitro assays of ADATs with yeast extracts and dsRNA substrates did not reveal any activity on mRNA (Gerber et al. 1998). However, none of the three ADAT genes in *F. graminearum* were specifically expressed during sexual reproduction. It is possible that fungi have ADAT-interacting proteins that are specifically expressed during sexual reproduction, and they form protein complexes with ADATs for A-to-I editing of mRNA. The other possibility is that the ADAT enzymes responsible for mRNA editing in *F. graminearum* are subjected to stage-specific phosphorylation or other posttranslational modifications for activation.

In animals, the ADARs bind to any dsRNA without apparent sequence specificity but perform A-to-I editing specifically within certain dsRNA substrates (Gott and Emeson 2000; Nishikura 2010). Although target recognition by metazoan ADARs and the mechanisms of substrate interaction are not well understood, it has been shown that the A's targeted for RNA-editing are embedded in an RNA stem (Wahlstedt and Öhman 2011; Barraud and Allain 2012). Furthermore, both nucleotides surrounding the edited adenosines and secondary structure elements affect the specificity and efficiency of RNA editing. In this study, we found that the 5′ − 1 preference in *F. graminearum* is U, and the 3′ + 1 preference is A or G, which is different from that of human ADAR and ADARB1 (Eggington et al. 2011). We also identified additional base preference beyond the nearest neighbor nucleotides in both the 5′ and 3′ positions, particularly, in the 3′ positions, suggesting that the sequence preferences of A-to-I editing is more specific in *F. graminearum.*

Regarding structure selectivity, unlike targeting A's embedded in RNA stems in animals (Barraud and Allain 2012), A-to-I editing preferentially targets A's in hairpin loops and the stability of hairpin loops affect editing efficiency in *F. graminearum*. In animals, the neighbor preferences of ADARs are mainly dictated by the catalytic domain, whereas selectivity derives mainly from the dsRBDs domain (Stephens et al. 2004; Eggington et al. 2011). *F. graminearum* lacks ADAR orthologs, but it has three ADATs. Interestingly, the anticodon loop of tRNA targeted by ADATs is a hairpin loop. The similar sequence-preference but distinct structure-selectivity of editing between *F. graminearum* and animals, indicative of the potential evolutionary link between mRNA editing and ADATs in fungi. Therefore, the A-to-I editing events identified in fungi together with their stage-specific roles in sexual reproduction provide great resources to study the functions, regulatory mechanisms, and evolution of RNA editing.

## Methods

### Library construction and sequencing

Genomic resequencing and strand-specific RNA-seq libraries were prepared with the wild-type strain PH-1 that was sequenced at the Broad Institute (Cuomo et al. 2007). Experimental methods, including sample collection, library construction, and sequencing, are described in detail in the Supplemental Methods. For each library, at least 30 million high quality reads were obtained.

### Read mapping and identification of RNA editing sites

RNA-seq reads of *F. graminearum* were aligned to the complete genome of PH-1 (King et al. 2015) available in Ensembl Fungi with program HISAT v 0.1.6-beta (Kim et al. 2015). RNA editing sites were identified by CLC Genomics Workbench 7.5 (CLC Bio), and a series of stringent filters were implemented to eliminate false positives as described in the Supplemental Methods. The methods used to analyze functional consequences and sequence and structure features of A-to-I editing sites were also summarized in the Supplemental Methods.

### Functional studies of genes

Experimental methods for targeted deletion of the five genes with *PUK1*-like editing events (Supplemental Table 4) and the *FgTAD1* gene, and generation of the *PUK1*^TGGTGG^ and *PUK1*^TAATAA^ constructs and transformants were described in the Supplemental Methods.

## Data access

Sequencing data from this study have been submitted to the NCBI Sequence Read Archive (SRA; http://www.ncbi.nlm.nih.gov/sra/) under accession numbers SRP062731 and SRP067538.

## Supplementary Material

Supplemental Material
