# Supplementary material for: Genome-wide A-to-I RNA editing in fungi independent of ADAR enzymes
Source: Genome Res. 2016 Apr;26(4):499–509. doi: 10.1101/gr.199877.115 (PMC4817773; doi:10.1101/gr.199877.115)
Supplement: Supplemental Material [file supp_26_4_499__index.html]

Genome-wide A-to-I RNA editing in fungi independent of ADAR enzymes — Genome-wide A-to-I RNA editing in fungi independent of ADAR enzymes — Supplemental Material 

# Genome-wide A-to-I RNA editing in fungi independent of ADAR enzymes

## Supplemental Material

**Files in this Data Supplement:**

- Supplemental Figures.pdf
- Supplemental Methods.pdf
- Supplemental Table 1.xlsx
- Supplemental Table 2.xlsx
- Supplemental Table 3.xlsx
- Supplemental Table 4.xlsx
