# Supplementary material for: Genome-wide A-to-I RNA editing in fungi independent of ADAR enzymes
Source: Genome Res. 2016 Apr;26(4):499–509. doi: 10.1101/gr.199877.115 (PMC4817773; doi:10.1101/gr.199877.115)
Supplement: Supplemental Material [file supp_gr.199877.115_Supplemental_Figures.pdf]

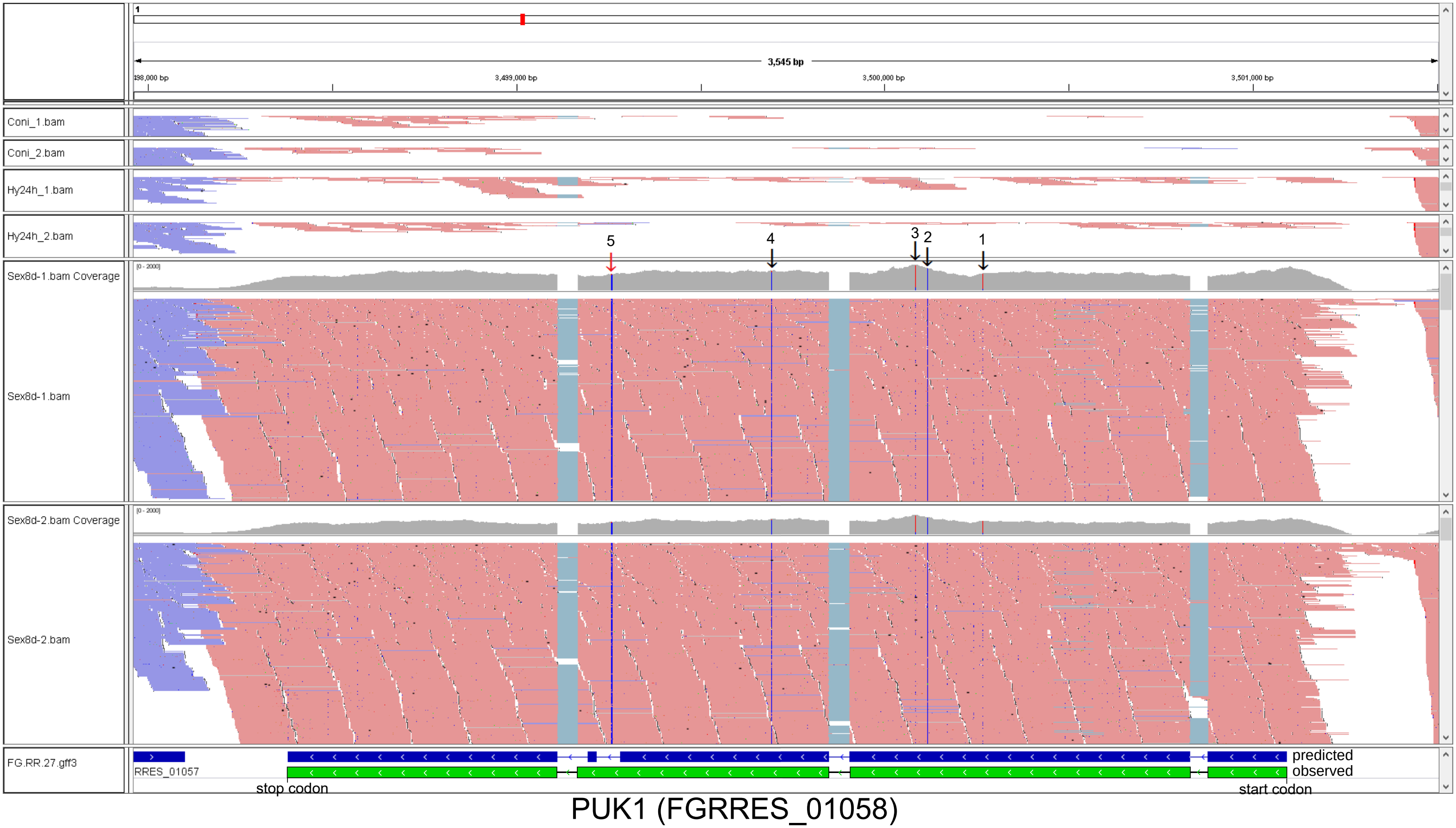

Supplemental Figure 1. IGV snapshot of RNA-seq reads mapped to the genomic region of *PUK1* in *F. graminearum*. RNA-seq reads from two biological replicates of conidia (Coni_1 and Coni_1), 24 h hyphae (Hy24h_1 and Hy24h_2), and 8-dpf perithecia (Sex8d-1 and Sex8d-2) matched to the 2.7-kp *PUK1* genomic region. Blue, reads transcribed from the sense-strand; red, reads transcribed from the antisense-strand. The third intron in the predicted gene model (bottom panel) is actually part of exon 3 in the cDNA. Five A-to-I editing sites (arrows 1-5) were identified in perithecium samples but none of them were found in conidia and hyphae samples. Red arrow (arrow 5) marks the site where UA^{1831}G UA^{1834}G to UG^{1831} GUG^{1834}G editing occurred. Editing levels are over 90% at sites 2, 4, and 5 but relatively low at sites 1 and 3.

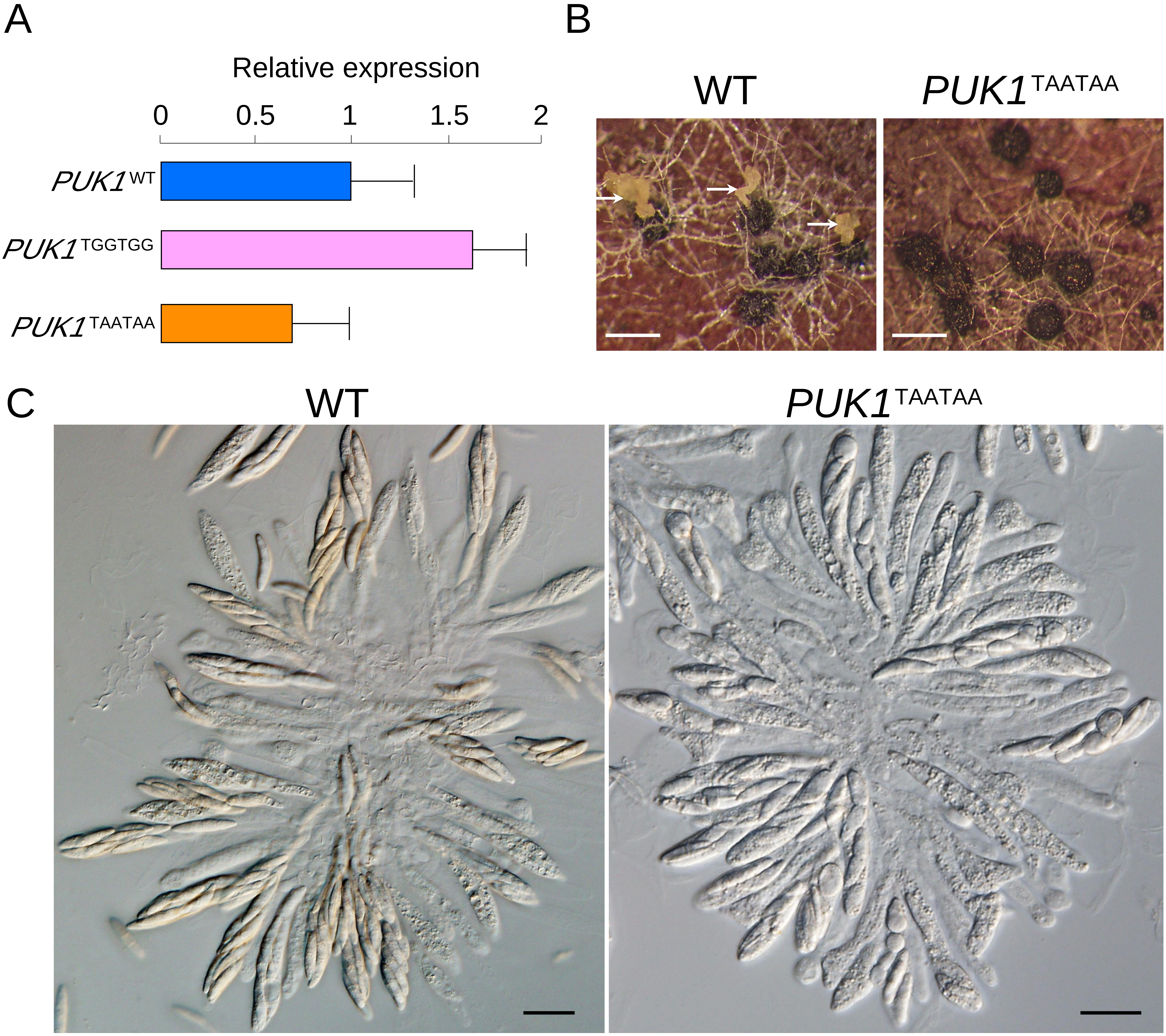

Supplemental Figure 2. Assays for the expression and function of *PUK1* mutant alleles. (*A*) Expression levels of the wild-type *PUK1* (*PUK1*^{WT}), *PUK1*^{TGGTGG}, and *PUK1*^{TAATAA} alleles in vegetative hyphae were assayed by qRT-PCR. The expression level of *PUK1*^{WT} was arbitrarily set to 1. Error bar indicates standard deviation (SD) calculated from data of three replicates. (*B*) Two-week-old mating cultures of the wild type (PH-1) and the *puk1*/*PUK1*^{TAATAA} transformant (*PUK1*^{TAATAA}) were examined for cirrhus production (marked with arrows) on perithecia. (*C*) Asci and ascospores formation were examined with perithecia from mating cultures 10 days post-fertilization. Abnormal ascospores (marked with arrows) were observed in the *PUK1*^{TAATAA} transformant. White bar = 1 mm, black bar = 20 μm.

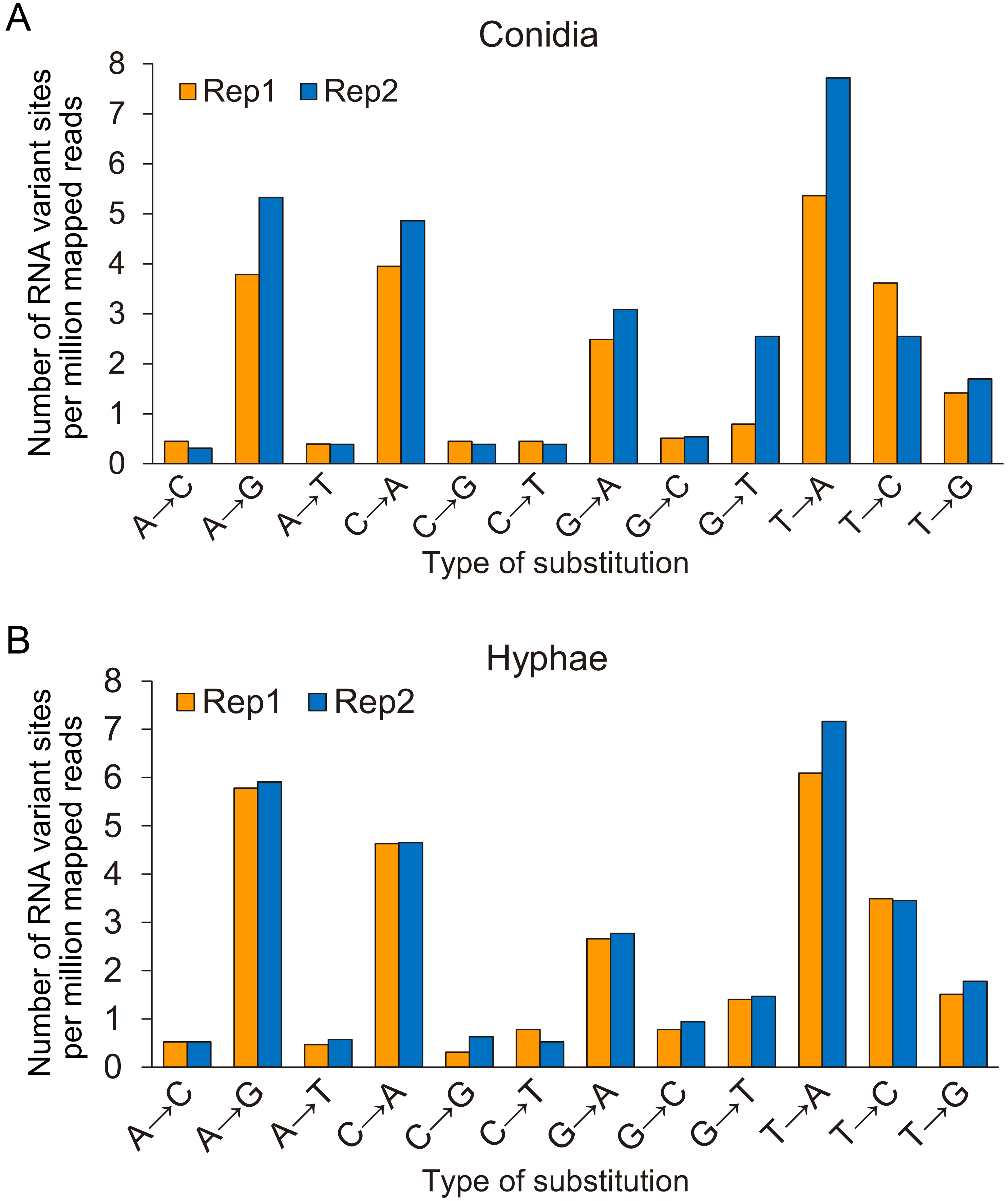

Supplemental Figure 3. Numbers of RNA variant sites per million mapped unduplicated reads identified in RNA-Seq data of conidia (*A*) and hyphae (*B*) for each of the 12 substitution types. Rep1 and Rep2 are results from two independent biological replicates. No enrichment for the A-to-G variants is observed in RNA-Seq data of conidia and hyphae.

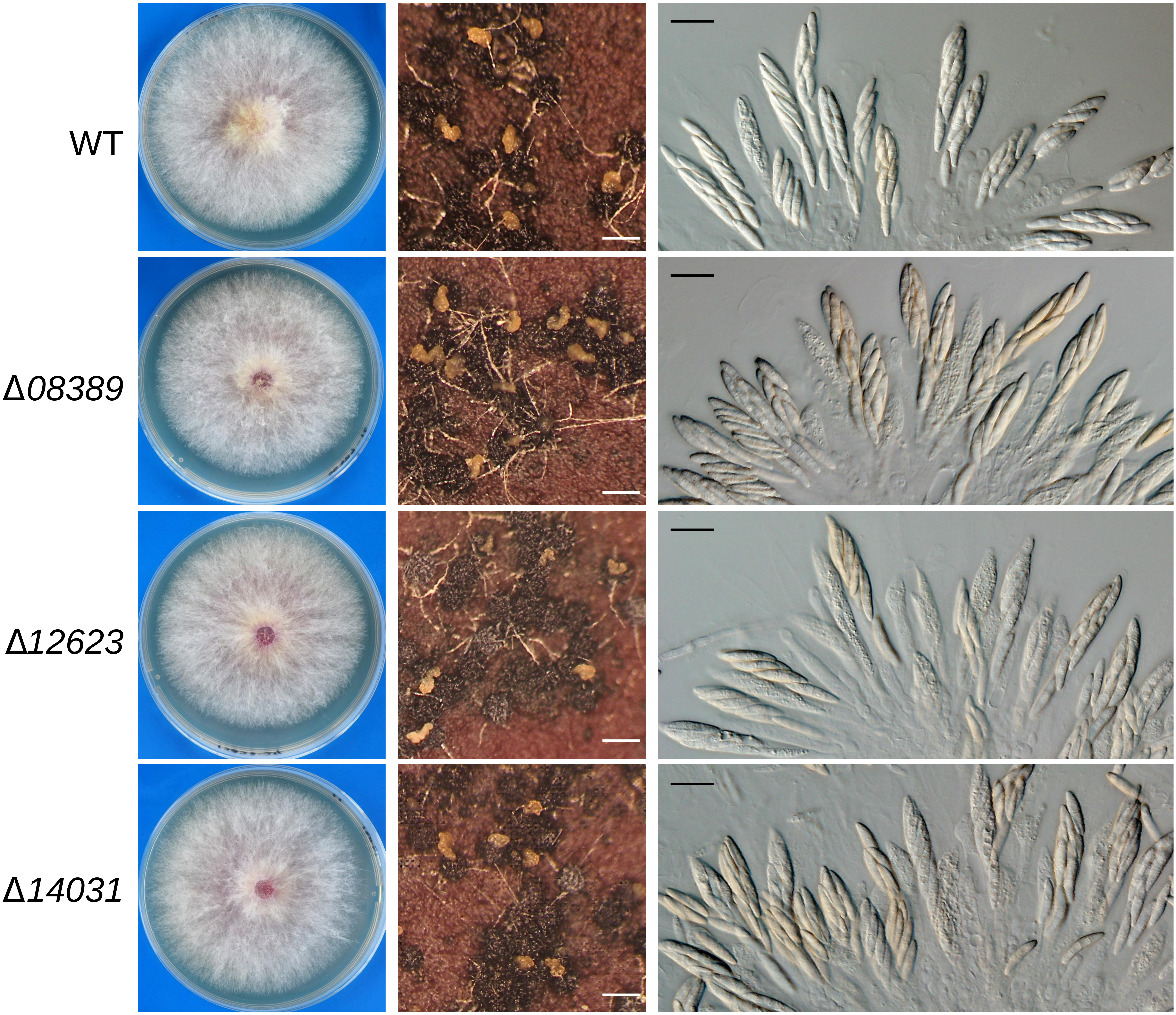

Supplemental Figure 4. Assays for colony morphology and sexual reproduction in mutants deleted of three genes with *PUK1*-like editing events. PDA (left panels) and mating (middle panels) cultures of the wild type (PH-1) and FGRRES_08389 (Δ*08389*), FGRRES_12623_M (Δ*12623*), and FGRRES_14031_M (Δ*14031*) deletion mutants were examined for colony morphology and the production of perithecia and cirrhi. Asci and ascospore formation (right panels) were examined with perithecia of the same set of strains. White bar = 1 mm, black bar = 20 µm.

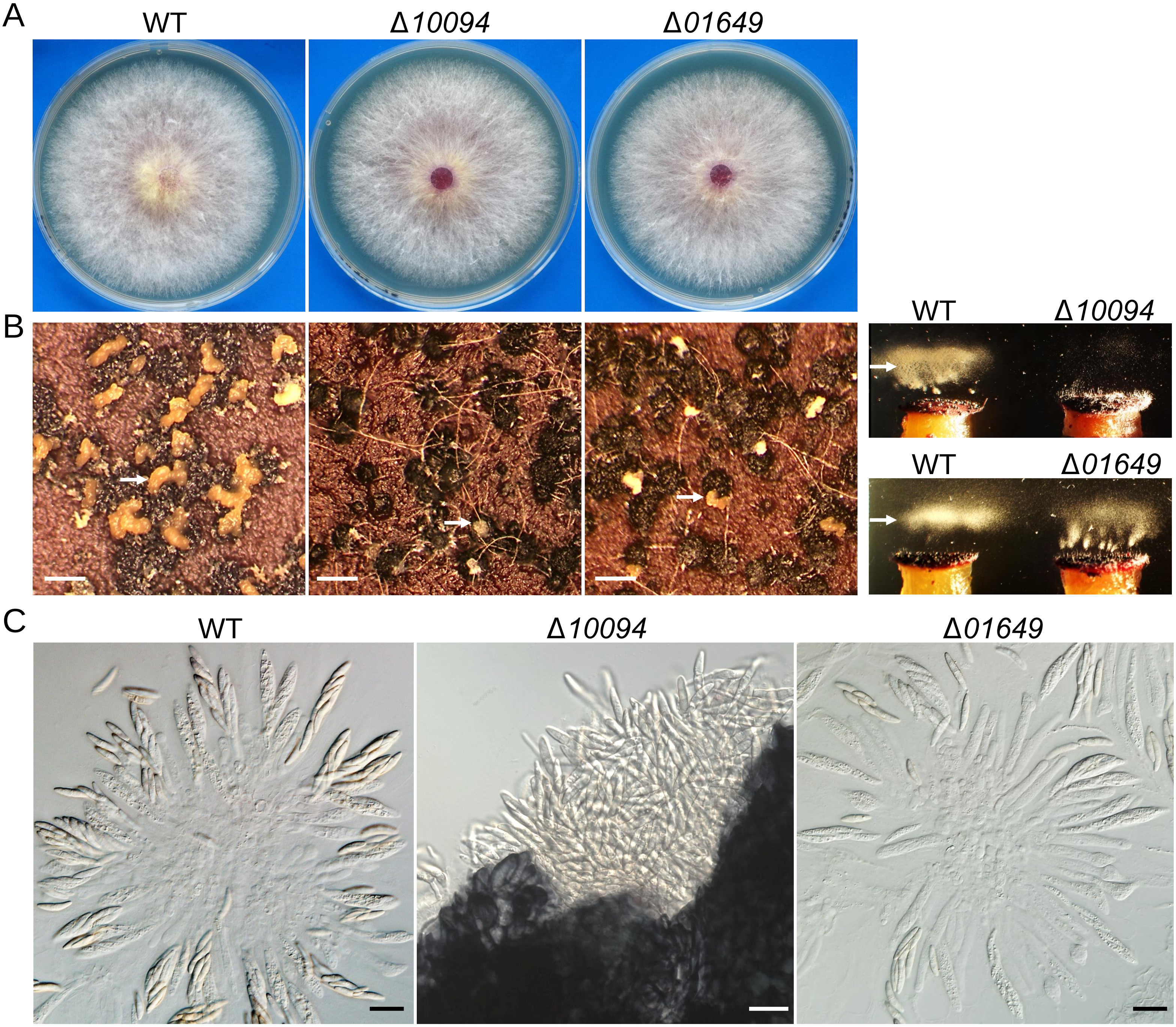

Supplemental Figure 5. Assays for defects in sexual reproduction in the FGRRES_10094 and FGRRES_01649 deletion mutants. The wild type PH-1 (WT) and deletion mutants of FGRRES_10094 (Δ*10094*) and FGRRES_01649 (Δ*01649*) were examined for colony morphology with PDA cultures (*A*), and cirrhus production and ascospore release with mating cultures (*B*). Arrows point to cirrhi (ascospores oozed from perithecia) and ascospore masses ejected from perithecia. Bar = 1 mm. (*C*) Asci and ascospores formed by PH-1 and the Δ*10094* and Δ*01649* mutants. Bar = 20 μm.

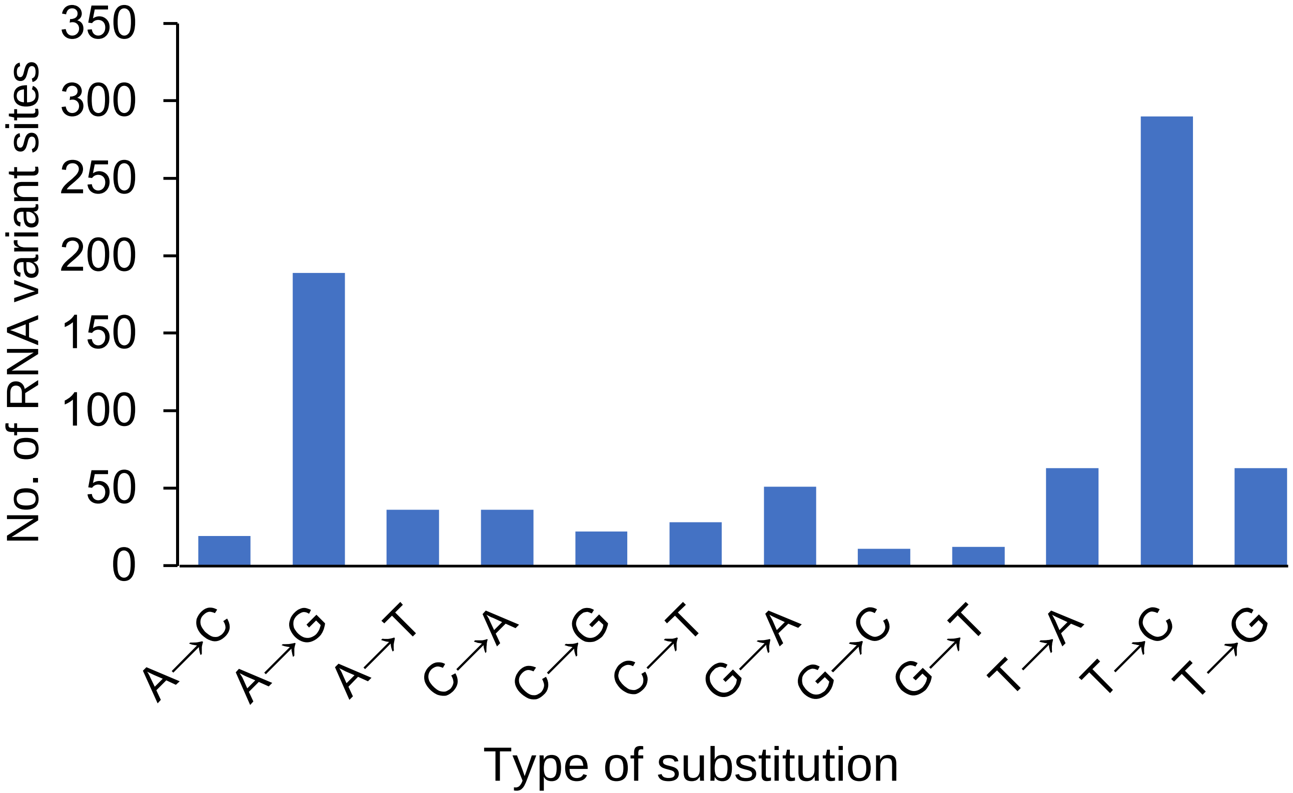

Supplemental Figure 6. Number of labelled RNA variant sites identified in published RNA-Seq data of hyphae in *Fusarium verticillioides* downloaded from the NCBI SRA database under accession numbers GSE61865 (Sikhakolli et al. 2012).
