# Supplementary material for: Genome-wide A-to-I RNA editing in fungi independent of ADAR enzymes
Source: Genome Res. 2016 Apr;26(4):499–509. doi: 10.1101/gr.199877.115 (PMC4817773; doi:10.1101/gr.199877.115)
Supplement: Supplemental Material [file supp_gr.199877.115_Supplemental_Methods.pdf]

Supplemental Methods

Culture conditions and phenotype assays: The wild-type strain PH-1 (Cuomo et al. 2007) and its derived transformants or mutants were routinely cultured on potato dextrose agar (PDA) plates at 25°C for growth and in carboxymethyl cellulose (CMC) medium (Cappellini and Peterson 1965) for conidiation as described (Zhou et al. 2010). For self-fertilization, cultures were grown on carrot agar plates (Leslie et al. 2006) for 7 days before knocking down aerial hyphae with sterile 0.1% Tween-60 solution as described (Luo et al. 2014). Perithecium formation, ascus development, and cirrhus production were assayed after incubation at 25°C for 1-2 weeks after fertilization under black light (Hu et al. 2014; Jiang et al. 2015).

Library construction and sequencing: For RNA-seq analysis, conidia collected from 5-day-old CMC cultures, vegetative hyphae harvested from 24 h liquid YEPD cultures, and perithecia collected from mating cultures 8 days post-fertilization (dpf) of strain PH-1 (Cuomo et al. 2007) were used for RNA isolation and purified with the RNAprep Pure Plant Kit (Tiangen Biotech, Beijing, China). For each tissue, RNA was isolated from two independent biological replicates. Contaminating rRNA was removed with the Ribo-Zero rRNA Removal Kit (Human/Mouse/Rat) (Catalog #: MRZH116, Illumina, USA). Strand-specific RNA-seq libraries were prepared with the NEBNext® Ultra™ Directional RNA Library Prep Kit (NEB, USA) and sequenced with Illumina HiSeq® 2500 at the 2×150 bp paired-end read mode. To exclude potential genomic variants of the PH-1 strain maintained in our lab, we also sequenced its genome with genomic DNA isolated from perithecia. The genomic DNA sequencing library was prepared with the NEBNext® Ultra™ DNA Library Prep Kit for Illumina® (NEB, USA) following the manufacturer's instruction and sequenced with the Illumina HiSeq® 2500 System, with a 2×101 bp paired-end read mode. Low quality reads and reads containing adapter and/or poly-N were removed from raw sequencing data with Trimmomatic (Bolger et al. 2014). For each library, at least 30 million high quality reads were obtained.

Read mapping and single-nucleotide variant calling: RNA-seq reads were aligned to the reference genome (King et al. 2015) downloaded from Ensembl Fungi (http://fungi.ensembl.org/Fusarium_graminearum/Info/Index) using program HISAT v 0.1.6-beta (Kim et al. 2015) with its two-step algorithm. The mapped strand-specific RNA-seq reads were divided into sense-strand and antisense-strand groups, respectively. DNA-seq reads were aligned using CLC Genomics Workbench 7.5 (CLC Bio, Denmark). SNV was called separately for the sense-strand and antisense-strand groups, and for the genome sequencing reads using the low frequency variant detection tool of CLC Genomics

Workbench 7.5, which employs statistical models for estimating the sequencing error rate with the default setting of 1% for required significance parameter.

To exclude amplification bias by PCR during library preparation, duplicate reads with identical start and end positions were removed from the read mappings by the duplicate mapped read removal plugin of CLC Genomics Workbench 7.5. The following filters were used to eliminate false-positives due to amplification bias, sequencing errors, and mapping errors: i) *Read filter* to remove non-specific matched reads likely related to erroneous mapping; ii) *Coverage and count filters* to retain only SNVs present in at least 5 reads with a minimum frequency of 3% and minimum coverage of 10 reads; iii) *Base quality filter* to remove reads with a central quality below 20 and neighborhood quality below 15 within 5 neighborhood radius; iv) *Read direction and position filters* to remove SNVs whose relative read direction and read position distribution are significantly different from the expected at the significance cut-off of 1%; and v) *Multiple type of mismatches filter* to discard SNV sites with more than one types of mismatch.

Identification of A-to-I editing sites in perithecia of *F. graminearum*: Consistent with the fact that the reference genome is of high quality and the same strain was used in this study, only 160 SNVs were identified in genome re-sequencing data of strain PH-1 (Cuomo et al. 2007). Authentic RNA SNVs were obtained by filtering out any genomic SNVs from the RNA variant sets. In total, 23,041 and 19,764 SNV sites were identified in the two independent biological replicates of perithecia, Rep1 and Rep2, respectively. Among these SNVs, 22,578 in Rep1 and 19,261 Rep2 correspond to A-to-G transitions. A total of 17,613 A-to-G transitions are common in both replicates, accounting for 91.4% of the A-to-G sites in Rep2. Because A-to-G variants are highly concordant between Rep1 and Rep2, we combined read mappings from these two replicates to maximize the statistical power for identification of A-to-I editing sites. A total of 26,056 A-to-G variants (referred to A-to-I editing sites in this study) were identified in the combined datasets at an estimated false-discovery rate of 0.43%. The non A-to-G variants are much less abundant (463 and 503 for Rep1 and Rep2, respectively) and only 35.9% of them are common between Rep1 and Rep2, suggesting that they are not related to RNA editing.

Identification of genes specifically expressed or upregulated in perithecia: The number of reads (counts) aligned to each predicted gene of PH-1 was calculated by FeatureCounts (Liao et al. 2014) based on the RNA-seq mappings. Differential expression analysis of genes in perithecia compared to conidia and hyphae stages was performed with the edgeRun package (Dimont et al. 2015) using the UCexactTest function with the Benjamini and Hochberg's algorithm to control the false discovery rate (FDR). To filter out weakly expressed genes, only genes with a minimum expression level of 1 count per million (cpm) in

at least two RNA-seq libraries were included in the analysis. Genes with a FDR below 0.05 and fold-change greater than 2 were considered to be upregulated genes. Genes upregulated in perithecia with an expression value (cpm) larger than 1 in both replicates of perithecia but lower than 1 in the RNA-seq libraries of conidia and hyphae were considered to be specifically expressed in perithecia.

Analyses of sequence and structure features of A-to-I editing sites in *F. graminearum*: Mutation site annotation and functional consequence prediction was performed with the SnpEff (Cingolani et al. 2012) and Amino Acid Changes tool of the CLC Genomics Workbench 7.5. Blast2GO (www.blast2go.com) was used for gene functional annotation. RNA-seq mappings were viewed using Integrative Genomics Viewer (IGV) (Robinson et al. 2011). Sequence logo was generated with WebLogo 3 (Crooks et al. 2004). Two Sample Logo (Vacic et al. 2006) was used to estimate and visualize the differences between neighboring nucleotides of the A-to-I editing sites identified in this study and 30,000 A sites randomly chosen from the predicted cDNA sequences of the *F. graminearum* genome (King et al. 2015). Secondary structures of RNA sequences containing the edited A sites (30 bp upstream and 30 bp downstream from the edited As) were predicted with RNAFold (Lorenz et al. 2011). Statistical analysis was performed using R (R Core Team 2015). The *P* value less than 0.05, 0.01, 0.001, and 0.0001 was designated with one (*), two (**), three (***), and four (****) asterisks, respectively.

Analysis of A-to-I editing in *F. verticillioides* and *N. crassa*: The polyA-selected but un-stranded RNA-seq data of *F. verticillioides* (Sikhakolli et al. 2012) and *N. crassa* (Wang et al. 2014) were downloaded from the NCBI SRA database under accession numbers GSE61865 and GSE41484, respectively. For *F. verticillioides*, RNA-seq reads of hyphae (2 h after induction of the sexual cycle) and perithecia (4-dpf) samples were mapped to the genome sequence of strain 7600 (FV3) obtained from Broad institute (http://www.broadinstitute.org/). For *N. crassa*, RNA-seq reads of hyphae before the sexual crossing (N6-0 h) and 5-dpf perithecia (N6-120 h) samples were mapped to the genome sequence of *N. crassa* OR74A (NC12) obtained from Broad institute (http://www.broadinstitute.org/). Genome-scale identification of A-to-I editing sites in *F. verticillioides* used the method similar to that for *F. graminearum*. Since the RNA-seq reads are unpaired and only 36 bp in length, we did not attempt to remove duplicated reads from the RNA-seq mappings. Because the strain of *F. verticillioides* used to generate the RNA-seq data was different from the strain whose genome was sequenced (Sikhakolli et al. 2012), we removed all homozygous variant sites and sites with extreme degree of variation (>90%), which were most likely to be genomic variants. Also because the library preparation protocol used to generate RNA-seq data of *F. verticillioides* was non-strand specific, we inferred the type of substitution based on the strand

information of annotated genes. Variant sites without strand information were excluded from the final list.

Generation of the gene knockout mutants: The split-marker approach (Catlett et al. 2003) was used to generate the gene replacement constructs for the five genes with *PUK1*-like editing events (Supplemental Table 4) and the *FgTAD1* gene. Protoplasts of the wild-type strain PH-1 (Cuomo et al. 2007) were prepared and transformed with each gene replacement construct as described previously (Hou et al. 2002). For transformant selection, hygromycin B (Calbiochem, La Jolla, CA) was added to the final concentration of 250 mg/ml. Hygromycin-resistant transformants were screed by PCR and putative knockout mutants were confirmed by Southern hybridization analyses.

Generation of the *PUK1*^{TGGTGG} and *PUK1*^{TAATAA} constructs and transformants: To generate the *PUK1*^{TGGTGG} allele, the *PUK1* gene (including its 1.5 kb promoter region) was amplified by overlapping PCR with the TA^{1831}G TA^{1834}G to TG^{1831}G TG^{1834}G mutations introduced into the PCR primers. The resulting PCR products were cloned into *Xho*I-digested plasmid pFL2 (Zhou et al. 2011) by yeast gap repair (Bruno et al. 2004). The same approach was used to generate the *PUK1*^{TAATAA} allele with two stop codons (TAATAA) inserted right behind the two edited stop codons TA^{1831}G TA^{1834}G. The *PUK1*^{TGGTGG} and *PUK1*^{TAATAA} constructs rescued from Trp+ yeast transformants were confirmed by sequencing analysis and transformed into the *puk1* deletion mutant. Geneticin-resistant transformants expressing the *PUK1*^{TGGTGG} and *PUK1*^{TAATAA} constructs were identified by PCR. For qRT-PCR analysis, RNA samples were isolated from vegetative hyphae harvested from 24 h YEPD cultures of PH-1 and transformants expressing the *PUK1*^{TGGTGG} or *PUK1*^{TAATAA} allele with the TRIzol reagent (Invitrogen, Carlsbad, CA). For each sample, at least three biological replicates were analyzed to calculate mean and standard deviation.

Supplemental reference

Bolger AM, Lohse M, Usadel B. 2014. Trimmomatic: a flexible trimmer for Illumina sequence data. *Bioinformatics* **30**: 2114-2120.

Bruno KS, Tenjo F, Li L, Hamer JE, Xu JR. 2004. Cellular localization and role of kinase activity of PMK1 in *Magnaporthe grisea*. *Eukaryotic Cell* **3**: 1525-1532.

Cappellini R, Peterson J. 1965. Macroconidium formation in submerged cultures by a non-sporulating strain of *Gibberella zeae*. *Mycologia*: 962-966.

Catlett N, Lee B-N, Yoder O, Turgeon BG. 2003. Split-marker recombination for efficient targeted deletion of fungal genes. *Fungal Genetics Newsletter*: 9-11.

Cingolani P, Platts A, Wang le L, Coon M, Nguyen T, Wang L, Land SJ, Lu X, Ruden DM. 2012. A program

for annotating and predicting the effects of single nucleotide polymorphisms, SnpEff: SNPs in the genome of Drosophila melanogaster strain w1118; iso-2; iso-3. *Fly (Austin)* **6**: 80-92.

Crooks GE, Hon G, Chandonia JM, Brenner SE. 2004. WebLogo: a sequence logo generator. *Genome Res* **14**: 1188-1190.

Cuomo CA, Gueldener U, Xu JR, Trail F, Turgeon BG, Di Pietro A, Walton JD, Ma LJ, Baker SE, Rep M et al. 2007. The *Fusarium graminearum* genome reveals a link between localized polymorphism and pathogen specialization. *Science* **317**: 1400-1402.

Dimont E, Shi J, Kirchner R, Hide W. 2015. edgeRun: an R package for sensitive, functionally relevant differential expression discovery using an unconditional exact test. *Bioinformatics* **31**: 2589-2590.

Hou ZM, Xue CY, Peng YL, Katan T, Kistler HC, Xu JR. 2002. A mitogen-activated protein kinase gene (MGV1) in *Fusarium graminearum* is required for female fertility, heterokaryon formation, and plant infection. *Mol Plant Microbe In* **15**: 1119-1127.

Hu S, Zhou X, Gu X, Cao S, Wang C, Xu J-R. 2014. The cAMP-PKA pathway regulates growth, sexual and asexual differentiation, and pathogenesis in *Fusarium graminearum*. *Mol Plant-Microbe Interact* **27**: 557-566.

Jiang C, Zhang S, Zhang Q, Tao Y, Wang C, Xu J-R. 2015. *FgSKN7* and *FgATF1* have overlapping functions in ascosporogenesis, pathogenesis and stress responses in *Fusarium graminearum*. *Environ Microbiol* **17**: 1245-1260.

Kim D, Langmead B, Salzberg SL. 2015. HISAT: a fast spliced aligner with low memory requirements. *Nat Methods* **12**: 357-360.

King R, Urban M, Hammond-Kosack MC, Hassani-Pak K, Hammond-Kosack KE. 2015. The completed genome sequence of the pathogenic ascomycete fungus *Fusarium graminearum*. *BMC Genomics* **16**: 544.

Leslie JF, Summerell BA, Bullock S. 2006. *The Fusarium laboratory manual*. Wiley Online Library.

Liao Y, Smyth GK, Shi W. 2014. featureCounts: an efficient general purpose program for assigning sequence reads to genomic features. *Bioinformatics* **30**: 923-930.

Lorenz R, Bernhart SH, Honer Zu Siederdissen C, Tafer H, Flamm C, Stadler PF, Hofacker IL. 2011. ViennaRNA Package 2.0. *Algorithms Mol Biol* **6**: 26.

Luo Y, Zhang H, Qi L, Zhang S, Zhou X, Zhang Y, Xu J-R. 2014. FgKin1 kinase localizes to the septal pore and plays a role in hyphal growth, ascospore germination, pathogenesis, and localization of Tub1 beta-tubulins in *Fusarium graminearum*. *New Phytolog* **204**: 943-954.

R Core Team. 2015. R: A language and environment for statistical computing. R Foundation for Statistical Computing, Vienna, Austria. ISBN 3-900051-07-0, URL https://www.R-project.org.

Robinson JT, Thorvaldsdottir H, Winckler W, Guttman M, Lander ES, Getz G, Mesirov JP. 2011. Integrative genomics viewer. *Nat Biotechnol* **29**: 24-26.

Sikhakolli UR, Lopez-Giraldez F, Li N, Common R, Townsend JP, Trail F. 2012. Transcriptome analyses during fruiting body formation in *Fusarium graminearum* and *Fusarium verticillioides* reflect species life history and ecology. *Fungal Genet Biol* **49**: 663-673.

Vacic V, Iakoucheva LM, Radivojac P. 2006. Two Sample Logo: a graphical representation of the differences between two sets of sequence alignments. *Bioinformatics* **22**: 1536-1537.

Wang Z, Lopez-Giraldez F, Lehr N, Farre M, Common R, Trail F, Townsend JP. 2014. Global Gene Expression and Focused Knockout Analysis Reveals Genes Associated with Fungal Fruiting Body Development in *Neurospora crassa*. *Eukaryot Cell* **13**: 154-169.

Zhou X, Heyer C, Choi YE, Mehrabi R, Xu JR. 2010. The CID1 cyclin C-like gene is important for plant infection in *Fusarium graminearum*. *Fungal Genet Biol* **47**: 143-151.

Zhou X, Li G, Xu JR. 2011. Efficient approaches for generating GFP fusion and epitope-tagging constructs in filamentous fungi. *Methods Mol Biol* **722**: 199-212.
